# Rational Electrolyte Structure Engineering for Highly Reversible Zinc Metal Anode in Aqueous Batteries

**DOI:** 10.1007/s40820-025-01950-7

**Published:** 2026-01-06

**Authors:** Yi Zhuang, Yukai Liang, Wenyao Zhang, Yuntong Sun, Zhenxing Wang, Jingyan Guan, Boyuan Zhu, Junjie Cui, Jiahao Tang, Jong-Min Lee, Junwu Zhu

**Affiliations:** 1 Key Laboratory for Soft Chemistry and Functional Materials Ministry of Education, Nanjing University of Science and Technology, Nanjing, 210094 People’s Republic of China; 2 School of Chemistry, Chemical Engineering and Biotechnology, Nanyang Technological University, 62 Nanyang Drive, Singapore, 637459 Singapore; 3 Department of Energy Science and Engineering, Daegu Gyeongbuk Institute of Science and Technology (DGIST), Daegu, 42988 Republic of Korea

**Keywords:** Aqueous zinc-ion batteries, Electrolyte structure, Anode/electrolyte interphase, Zinc anode

## Abstract

Aqueous zinc-ion batteries (AZIBs) have garnered considerable attention as promising post-lithium energy storage technologies owing to their intrinsic safety, cost-effectiveness, and competitive gravimetric energy density. However, their practical commercialization is hindered by critical challenges on the anode side, including dendrite growth and parasitic reactions at the anode/electrolyte interface. Recent studies highlight that rational electrolyte structure engineering offers an effective route to mitigate these issues and strengthen the electrochemical performance of the zinc metal anode. In this review, we systematically summarize state-of-the-art strategies for electrolyte optimization, with a particular focus on the zinc salts regulation, electrolyte additives, and the construction of novel electrolytes, while elucidating the underlying design principles. We further discuss the key structure–property relationships governing electrolyte behavior to provide guidance for the development of next-generation electrolytes. Finally, future perspectives on advanced electrolyte design are proposed. This review aims to serve as a comprehensive reference for researchers exploring high-performance electrolyte engineering in AZIBs.

## Highlights

- This review systematically summarizes the electrochemical principles governing Zn^2+^ nucleation and deposition, elucidating their intrinsic correlations.
- The review discusses zinc salt optimization, electrolyte additives, and novel electrolyte designs, providing mechanistic insights into anodic Zn^2+^ electrodeposition.
- The review proposes future directions for aqueous zinc metal anode, including dynamic reconstruction, AI-guided additive screening, etc.

## Graphical abstract

**Figure:**
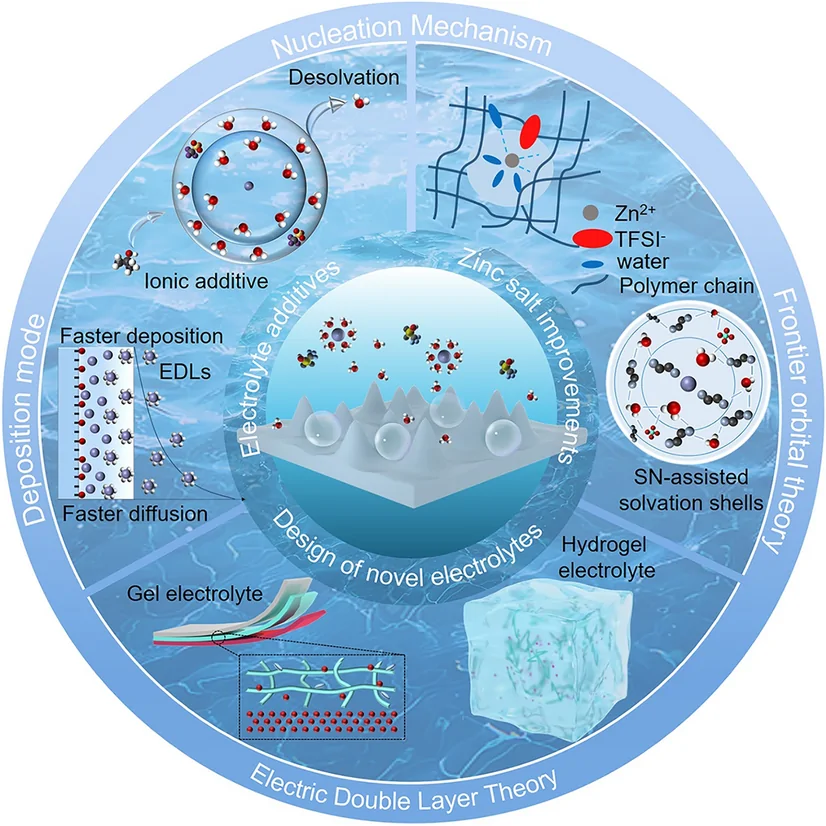

## Introduction

The rapid depletion of fossil fuels and escalating environmental concerns have intensified the demand for advanced energy storage technologies, making their development a critical global priority [1]. Among sustainable energy solutions, rechargeable batteries are pivotal, providing vital energy storage and conversion for electronic devices and transportation systems, playing an indispensable role in the pursuit of carbon neutrality.

Aqueous electrolytes offer ionic conductivities that are two orders of magnitude higher than the organic counterparts and, importantly, exhibit outstanding intrinsic safety. Therefore, aqueous metal-ion batteries are emerging as high-safety candidates for large-scale energy storage applications. In particular, aqueous zinc-ion batteries (AZIBs), using zinc ions (Zn^2+^) as charge carriers, have attracted considerable attention due to their low redox potential (− 0.76 V vs. standard hydrogen electrode, SHE), high theoretical capacity (5855 mAh cm⁻^3^ and 820 mAh g⁻^1^), intrinsic safety, and facile processing [2–4]. Revisiting the Zn^2+^ storage mechanism based on Zn^2+^/Zn redox couple (Zn^2+^  + 2e^−^  ⇌ Zn), the interfacial zinc chemistry at the electrode/electrolyte interface plays a critical role, especially for the metallic Zn anode. In neutral electrolytes, solvated Zn^2+^ forms a stable hydration shell with abundant polar water molecules, while water molecules preferentially adsorb onto the Stern layer of the electric double layer (EDL), leading to parasitic water reduction and increased internal pressure. The associated hydrogen evolution reaction (HER) fluctuates the pH value in local areas, enriching anions (e.g., OH^−^, SO_4_^2−^), which subsequently undergo complexation and generate insulating and passivating zinc byproducts. These processes continuously consume active Zn^2+^ and hinder ion/electron transport [5–7]. Furthermore, the uncontrolled growth of zinc dendrites, arising from the non-uniform Zn^2+^ flux, desolvation, and nucleation barriers, remains a crucial obstacle, similar to other (alkali) metal anodes. The issues of HER, passivation, and dendrite formation are interrelated: dendrite growth enlarges the anode surface area and accelerates HER; HER elevates local OH^−^ concentration and promotes inert byproduct formation; these byproducts deposit unevenly, exacerbate electric field polarization, and further stimulate dendrite growth. These synergistic degradation leads to capacity fading, reduced Coulombic efficiency (CE), and shortened battery lifespan.

To address these challenges, tremendous efforts have been devoted to moderating the electrochemical behavior of Zn^2+^ and water molecules, including anode design [8], artificial interphase engineering [9–11], electrolyte structure regulation [12, 13], charging protocol optimization [14], etc. Among these, electrolyte engineering has emerged as a pivotal strategy for advancing Zn metal anode in AZIBs, owing to its profound influence on Zn^2+^ solvation structure, ion flux, migration dynamics, and nucleation behavior, and in situ formation of functional interphases. Early approaches focused on adjusting salt types and concentration to tailor Zn^2+^ solvation, thereby suppressing water-induced side reactions by weakening water activity. In addition to these effects, many additives promote in situ formation of protective interphases that isolate the reactive water molecules, offering ion sieving capabilities that modulate Zn^2+^ desolvation and enable uniform nucleation on the Zn anode, mitigating dendrite growth. Recently, a dynamic interphase concept [15] has been proven to achieve real-time conformal contact with the Zn anode, continuously regulating deposition behavior and enhancing long-term anode stability. Beyond this, a range of emerging concepts and theoretical frameworks have further advanced the landscape of electrolyte design.

**Fig. 1:**
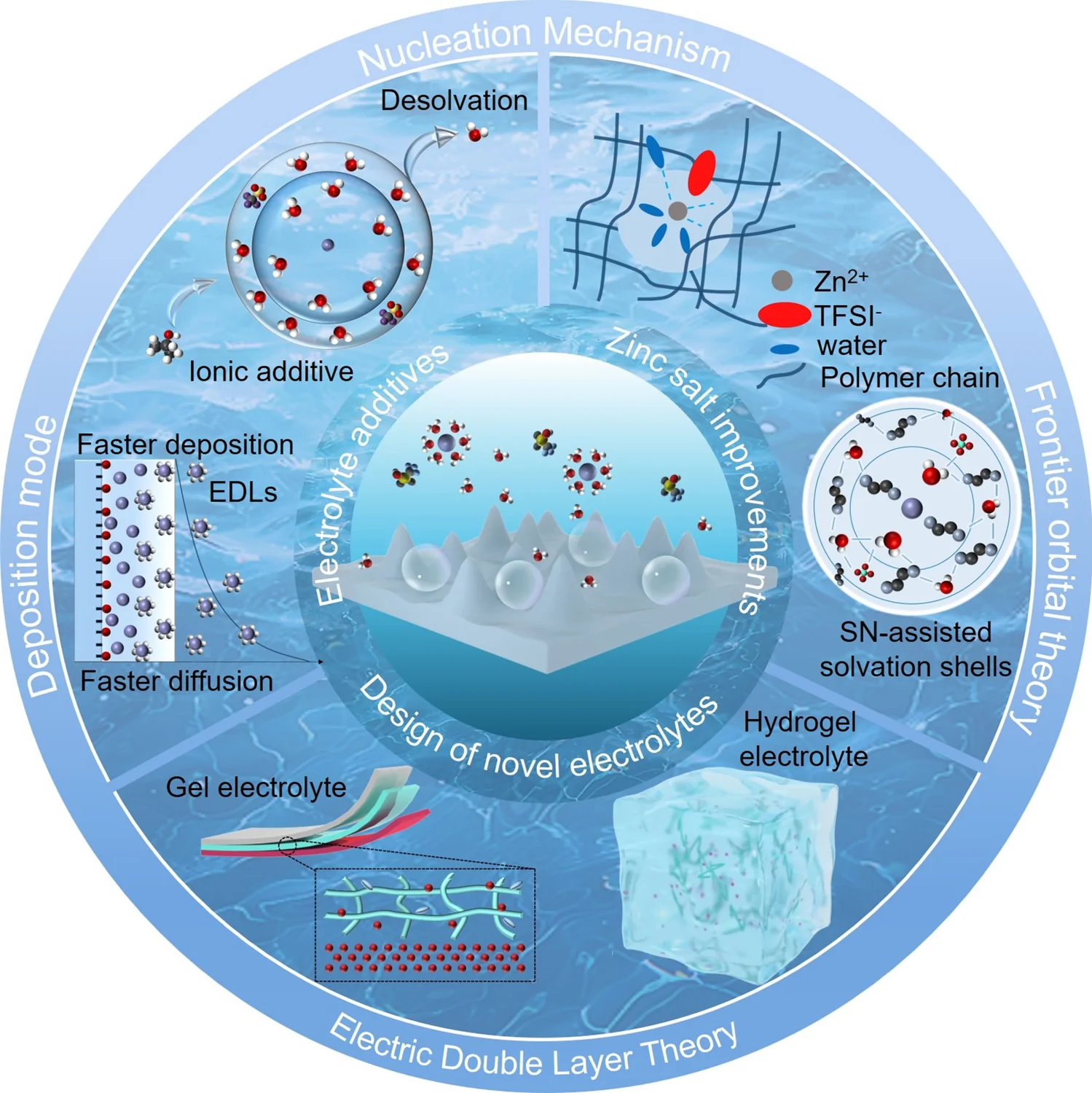
Electrolyte structure regulation strategies for Zn metal anodes in AZIBs

In this review, we systematically summarize the electrochemical principles governing Zn^2^⁺ nucleation and deposition, while elucidating their intrinsic interrelationships. Adopting a chronological framework, we discuss key developments in (i) zinc salt optimization, (ii) functional electrolyte additives, and (iii) the design of novel electrolyte systems (Fig. 1), providing mechanistic insights into Zn^2^⁺ electrodeposition from the perspective of electrolyte engineering. In contrast to prior reviews that classify additives by chemical composition, we organize the additive section based on dominant functional mechanisms, including electrostatic shielding, interfacial adsorption, desolvation modulation, in situ solid electrolyte interphase (SEI) formation, and crystal-plane engineering. These mechanisms are critically evaluated to reveal the fundamental processes underlying improved electrochemical performance. Furthermore, we highlight recent advances in dynamic interfacial construction, emphasizing real-time, self-regulating stabilization strategies under cycling conditions. Finally, we summarize the recent progress, existing challenges, and provide prospects in electrolyte engineering for Zn metal anode in AZIBs, aiming to inspire new insights and accelerate their practical deployment.

## Electrochemical Theory of Zn^2+^ Nucleation and Growth

The electrochemical performance of Zn metal anode in AZIBs is critically influenced by the nucleation particle size and nucleation energy barrier, two key parameters that dictate Zn^2+^ deposition behavior and consequently affect both cycling stability and battery lifespan. The EDL model provides an effective theoretical framework for elucidating the nucleation and growth mechanisms at the anode/electrolyte interface. In conventional ZnSO_4_ electrolytes, solvated Zn^2+^ ions, primarily in the form of [Zn(H_2_O)_6_]^2+^, diffuse from the diffusion layer to the outer Helmholtz plane (OHP) and undergo desolvation under long-range electrostatic interactions. The energy barrier associated with this desolvation process governs the kinetics of Zn^2+^ deposition [16]. Additionally, the potential drop across the Helmholtz layer serves as a crucial descriptor for nucleation and growth, influencing both the critical nucleation size and nucleation rate. The electric field distribution within the EDL further modulates Zn^2+^ deposition morphology [17]. Therefore, an in-depth understanding of these descriptors and interfacial parameters from an electrochemical theory perspective is essential for rationalizing Zn^2+^ nucleation behavior and guiding the design of high-performance AZIBs.

### Electric Double Layer Theory

**Fig. 2:**
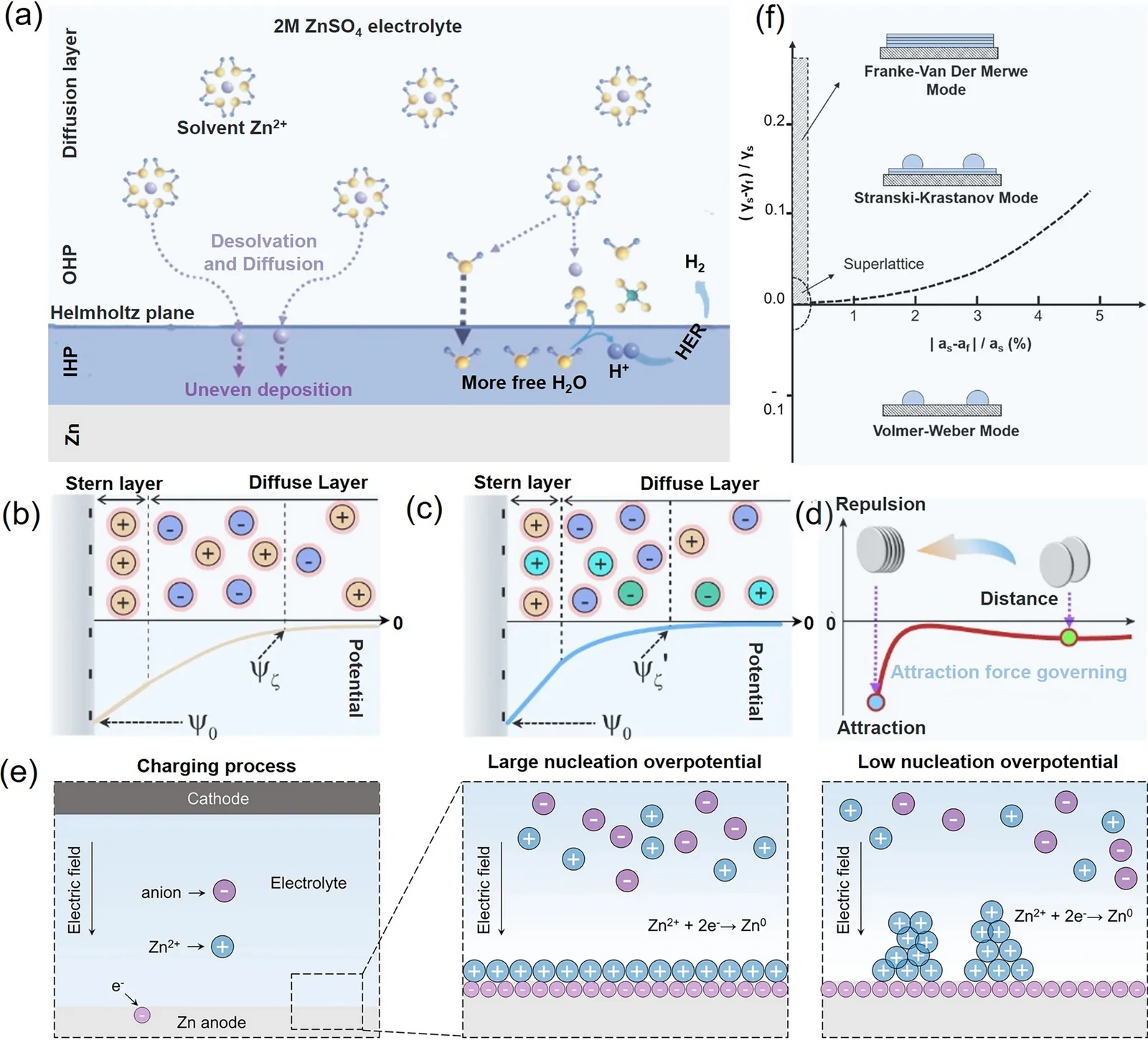
**a** Schematic illustration of Zn deposition process [18]. Comparison of the EDL structures of Zn deposits in **b** ZnSO_4_ and** c** La^3+^-ZnSO_4_ electrolytes [21]. **d** Proposed growth models of Zn deposits in La^3+^-ZnSO_4_ electrolytes [21]. **e** Schematic diagram of the effect of overpotential on Zn deposition behavior [28]. **f** Three nucleation regions mapped as a function of lattice mismatch (x-axis) and surface energy differences (y-axis), where γ_s_ and γ_f_ represent the surface energies of the substrate and deposited film, respectively, and a_s_ an a_f_ denote their corresponding lattice parameters [23, 30]

To elucidate the spatial arrangement of ions and solvent molecules at the anode/electrolyte interface, the EDL theory provides a fundamental framework for understanding interfacial reactions and performance-enhancing mechanisms in AZIBs. According to the Stern model, the EDL in AZIBs is composed of two distinct regions: the Stern layer and the diffusion layer. The Stern layer comprises the inner Helmholtz plane (IHP) and the outer Helmholtz plane (OHP) [16]. The IHP contains specifically adsorbed ions directly adjacent to the electrode surface, whereas the OHP host partially desolvated Zn^2^⁺ ions and a portion of the anions. Beyond the OHP lies the diffusion layer, extending into the bulk electrolyte. During deposition, Zn^2+^ migrates from the diffusion layer to the OHP, undergoes desolvation, and is subsequently reduced and deposited within the IHP(Fig. 2a) [18].

Hydrogen evolution and by-product formation, two key factors affecting the cycling stability and long-term performance of Zn anodes, are closely linked to the EDL, whose structure and composition critically govern interfacial reactivity [16]. Taking the ZnSO_4_-based electrolyte as a representative example, hydrated Zn^2+^ ions [Zn(H_2_O)_6_]^2^⁺ migrate from the bulk solution into the EDL during charging. Upon partial or complete desolvation, Zn^2^⁺ ions are reduced and electrodeposited onto the anode surface. However, incompletely desolvated species may reach the interface, triggering parasitic hydrogen evolution and corrosion of the zinc anode. Additionally, the preferential accumulation of water dipoles and sulfate anions (SO_4_^2^⁻) within the EDL intensifies electrostatic repulsion among adjacent Zn nuclei, disrupting uniform nucleation and resulting in dispersed, loosely packed platelet-like deposits [19]. The extent of electrostatic repulsion is determined by the EDL thickness (δ), which, for a planar interface, can be expressed analytically as a function of the Debye length (${\kappa }^{-1}$) and the dimensionless surface charge density (σ). The explicit form of δ is given by Eq. (1) [20]:

1 $$\delta =\frac{1}{\kappa }\mathrm{ln}\left(\frac{\mathrm{tanh}\left(\frac{{\mathrm{sinh}}^{-1}\frac{\sigma }{2}}{2}\right)}{\mathrm{tanh}\left(\frac{{\mathrm{sinh}}^{-1}\frac{\sigma }{2}}{200}\right)}\right)$$

Herein, tanh⁻^1^ and sinh⁻^1^ represent the inverse hyperbolic tangent and inverse hyperbolic sine functions, respectively.

A decrease in the EDL thickness *δ*, achieved by reducing the Debye length ${\kappa }^{-1}$ or the surface charge density σ, effectively migrates inter-platelet electrostatic repulsion and facilitates dense, uniform zinc electrodeposition. As a representative strategy, Qie et al. introduced La(NO_3_)_3_ into an aqueous electrolyte. The high-valent La^3^⁺ cations competitively replace Zn^2^⁺ at the interface, thereby decreasing the net surface charge of the nascent zinc layer. As shown in Fig. 2b–d, this charge attenuation leads to a significant contraction in δ, resulting in improved cycling stability and enhanced electrochemical performance of the cell [21].

### Nucleation Mechanism of Zn^2+^

#### Nucleation Barrier

Zinc deposition initiates with nucleation: Zn^2+^ ions first migrate toward the anode–electrolyte interface under the influence of an electric field and then nucleate on the Zn anode surface after overcoming the corresponding nucleation energy barrier [22]. A high nucleation barrier can limit the number of active nucleation sites, thereby promoting non-uniform zinc deposition (Fig. 2e). During continued deposition, Zn^2+^ ions undergo two-dimensional surface diffusion, which lead to Volmer–Weber growth characterized by island-like, inhomogeneous deposition. This uneven morphology disrupts the electric field distribution and ultimately induces the tip effect. As tip curvature increases, the local electric field intensity and surface charge density rise, enabling Zn^2+^ ions in the vicinity to overcome the nucleation barrier more readily and initiate dendritic growth. Moreover, ion concentration plays a vital role in the nucleation process: regions with higher ionic concentration or faster ion transport exhibit reduced nucleation barriers [23, 24]. Recent studies have demonstrated that both the binding energy between the Zn^2+^ and the anode substrate, and lattice adaptability significantly influence the energy barrier for nucleation.

To suppress dendrite formation, Zhao et al. introduced cyclodextrin (α-CD) as an electrolyte additive. The α-CD molecules interact strongly with metallic zinc, preferentially adsorbing onto the anode surface and lowering the nucleation barrier. This adsorption also induces additional surface charge on the zinc, generating enhanced electrostatic attraction for Zn^2+^ and guiding its deposition. Experimental results show that the nucleation overpotential on Zn foil decreases progressively with increasing α-CD concentration, confirming its effectiveness in reducing the zinc nucleation barrier. As a result, the α-CD additive promotes three-dimensional Zn^2+^ diffusion, leading to more uniform deposition [25].

#### Nucleation Particle Size

The nucleation particle size also plays a critical role in determining the electrochemical performance of Zn anodes. According to classical homogeneous nucleation theory, the critical radius (*r*) of the spherical nucleus is given by:

2 $$r=2\frac{\gamma {V}_{\mathrm{m}}}{F|\eta |}$$

where *γ* is the surface energy at the anode–electrolyte interface, *V*_m_ is the molar volume of Zn, *F* is the Faraday constant, and *η* is the nucleation overpotential. This expression indicates that smaller nucleation particle size corresponds to higher polarization overpotentials, which in turn enhance the nucleation driving force and promote finer, more uniform zinc deposition [18, 26]. Zhou et al. utilized ZnCl_2_-based electrolytes to investigate how electrolyte concentration influences the nucleation radius and Zn deposition behavior. In situ atomic force microscopy (AFM) observations revealed that in dilute electrolytes, large and sparsely distributed zinc nuclei were formed. In contrast, medium-concentration electrolytes minimized the *γ*/*η* ratio, leading to the formation of smaller zinc nuclei and enabling uniform, dense deposition of zinc species [27].

#### Nucleation Rate

The nucleation rate significantly influences the stability of the Zn anode, as a higher nucleation rate promotes more uniform Zn^2+^deposition during cycling. The nucleation rate (*ω*) can be described by Eq.:

3 $$\omega =K\text{ exp}\left(-\frac{\pi h{\sigma }^{2}LA}{\rho nFRT\eta }\right)$$

where *K* is the pre-exponential factor, σ is the interfacial tension, *L* is Avogadro number, n is the valence number of the metal ion, *R* and *F* are the gas constant and Faraday’s constant, *T* is the absolute temperature, and *ρ, h*, and *A* are the density, atomic height, and atomic weight of the deposited metal Zn, respectively. This expression indicates that the nucleation rate increases exponentially with the increase in overpotential, thereby allowing the formation of a uniform and compact Zn layer. Leveraging this principle, Hu et al. introduced sodium L-tartrate (Na-L) into the ZnSO_4_ solution to regulate the nucleation overpotential and improve zinc deposition uniformity. With the Na-L additive, the nucleation overpotential increased from 28.3 to 45.9 mV. Chronoamperometry measurements further revealed that Na-L enabled uniform three-dimensional diffusion of Zn^2+^, as evidenced by the rapid stabilization of the current density, suggesting homogeneous crystal growth during electrodeposition [28].

### Growth Mode of Deposits

The physicochemical properties of the substrate, particularly the lattice mismatch between zinc crystals and the substrate, directly affect its zincophilicity [29]. Accordingly, three classical deposition growth modes have been proposed based on the degree of lattice mismatch and interfacial energy differences between the deposit and the substrate: the Frank–Van der Merwe mode, the Volmer–Weber mode, and the Stranski–Krastanov mode (Fig. 2f) [5, 23, 30]. When the lattice mismatch is minimal, epitaxial growth favors smooth and continuous film deposition, characteristic of the Frank–Van der Merwe growth mode. As the mismatch increases, deposition behavior transitions to the Stranski–Krastanov mode, where layer-by-layer growth is followed by island formation. In cases of significant mismatch, where the interatomic forces within the deposited atoms surpass the adhesion to the substrate, the Volmer–Weber mode dominates, resulting in discrete island growth.

Based on this framework, regulating the lattice mismatch between the substrate and zinc enables controlled zinc nucleation and growth, leading to compact, dendrite-free deposits. For instance, Li et al. introduced maleic anhydride (MA) as an additive, which preferentially adsorbs on the (002) facet of Zn. This surface-selective interaction guides the deposition toward the Frank–Van der Merwe mode, thereby suppressing dendrite formation and enhancing uniformity [6]. Similarly, Ma et al. introduced sodium hyaluronate (SH) in ZnSO_4_ electrolyte. The SH exhibits a strong affinity for Zn, restructuring the electric double layer and forming a dynamic Zn-SH* interface. This promotes the preferential growth of Zn along the (002) plane, attributed to the polar functional groups in SH that improve surface adsorption. Moreover, the enhanced wettability of the SH-modified electrolyte facilitates Frank–Van der Merwe-type deposition, yielding a uniform and dense Zn deposition [31].

### Frontier Orbital Theory

In the development of electrolyte additives, the highest occupied molecular orbital (HOMO) and lowest unoccupied molecular orbital (LUMO) energy levels, derived from frontier molecular orbital theory, are crucial parameters for screening solvents and additives in electrolyte systems [32, 33]. A higher HOMO energy level enhances a molecule’s electron-donating ability, thereby facilitating coordination with metal ions or adsorption on electrode surfaces. In contrast, a lower LUMO energy level indicates a stronger electron-accepting ability, promoting the reduction and deposition of metal ions. Molecules that exhibit both low HOMO and high LUMO energies typically possess strong chemisorptive properties. Density functional theory (DFT) calculations of HOMO–LUMO levels are therefore widely employed to guide the rational selection of electrolyte components. The energy difference between HOMO and LUMO is closely related to both the electrochemical stability window and the electrode potential. Ideally, the electrode potential must lie within the HOMO–LUMO energy range of the electrolyte to prevent decomposition [34]. For instance, when the Fermi level of a cathode material is lower than the HOMO level of the electrolyte, electrons can transfer from the electrolyte to the cathode, leading to oxidation of the electrolyte. Hence, the working potentials of both anode and cathode must fall within the HOMO–LUMO gap of the electrolyte system to ensure stability [35, 36].

Additives with a narrow HOMO–LUMO gap can diminish interfacial charge-transfer resistance, thereby lowering the nucleation barrier for zinc deposition. For example, Wang et al. employed a system comprising Zn(BF_4_)_2_·4H_2_O salt and vinylene carbonate (VC) solution, where DFT calculations revealed a decreased HOMO–LUMO gap and elevated HOMO level in the solvated structure. VC exhibits a higher HOMO level at − 6.96 eV compared to water (− 8.06 eV), indicating enhanced electron-donating capability and improved charge transfer, thus increasing the reduction stability of the electrolyte [37]. Additionally, LUMO and HOMO levels are strongly associated with SEI formation. Additives with low LUMO levels preferentially accept electrons and are reduced at the electrode surface, facilitating SEI formation and uniform Zn^2^⁺ deposition [38]. Han et al. introduced tetradecafluorononane-1,9-diol (TDFND) as an additive, which, with a low LUMO of 0.10 eV significantly lower than that of water, underwent preferential reduction to form a ZnF_2_-rich SEI layer [39]. Li et al. employed sodium diethyldithiocarbamate (DDTC) and calculated the LUMO for H_2_O, Zn^2^⁺–H_2_O, DDTC⁻, and Zn–DDTC to be 2.06, − 0.75, 0.01, and − 1.88 eV, respectively. This indicates that DDTC coordinated with Zn^2⁺^ undergoes preferential reduction, contributing to SEI formation on the anode surface [40]. Similarly, Wei et al. utilized the HOMO energy levels to screen non-sacrificial additives for Zn anode stabilization. Among three anionic surfactants, sodium dodecyl benzene sulfonate (SDBS) possessed the highest HOMO level and strongest electron-donating capability, consistent with its high binding and adsorption energy. This strong coordination with Zn^2+^ helped reduce active water content and enhanced superior adsorption, thereby inhibiting Zn-H_2_O interactions [41]. Notably, LUMO and HOMO levels are key design parameters for cathode material modification. For instance, Ye et al. introduced − CN groups into the organic cathode material Hexaazatrinaphthalene, thereby lowering its LUMO and HOMO energies, increasing the operating voltage, narrowing the energy gap, and enhancing electronic conductivity and charge transport [32].

Nevertheless, it is important to recognize that in practical electrolytes, the electrochemical stability window cannot be solely determined by the HOMO–LUMO gap due to the influence of solvation effects and additives. Although the HOMO and LUMO levels are correlated with redox behavior, an energy offset of several eVs may occur. Therefore, caution should be exercised when using HOMO and LUMO as a direct indicator of the electrochemical stability [42].

## Electrolyte Structure Regulation

### Zinc Salt Improvements

The selection of zinc salt fundamentally determines the physicochemical properties of the aqueous electrolyte, directly impacting the electrochemical performance of Zn anode in AZIBs. Crucially, the nature of the salt governs electrolyte pH, which strongly influences Zn^2+^ electrodeposition behavior, alongside the parasitic HER kinetics and surface passivation. In detail, the anion size and chemical properties significantly affect the Zn^2^⁺ solvation structure, determining the number of coordinated water molecules, the energy barrier for Zn^2^⁺ desolvation, and the overall ion diffusion dynamics. The intrinsic water solubility of different zinc salts further constrains the maximum achievable salt concentration in the electrolyte. Notably, electrolyte concentration has a profound impact on solvation chemistry: higher concentrations can reconstruct solvation shells, reduce free water activity, increase viscosity, and modulate ion transport. Therefore, rational selection of zinc salt requires a comprehensive balance among key parameters, including pH, anion size, concentration, desolvation kinetics, and ion diffusion capabilities, to achieve reversible Zn plating/stripping and mitigate parasitic reactions.

Conventional zinc salts, such as ZnCl_2_, Zn(ClO_4_)_2_, Zn(NO_3_)_2_, Zn(CF_3_SO_3_)_2,_ and Zn(CH_3_COO)_2_, have been explored, each demonstrating distinct chemical properties and consequently differing impacts on electrolyte pH, solvation structure, and kinetics for the Zn anode (Table 1) [43–49], while these salts still face several challenges, including unstable Zn^2+^ solvation structures, high water reactivity, uneven ion flux, all of which might lead to the parasitic side reactions thereby reducing cycle life and coulombic efficiency. Initially, KOH was employed as the electrolyte due to the faster Zn^2+^ kinetics in alkaline media. However, long-term battery operation is hindered by anode passivation caused by self-corrosion and electrochemical corrosion. In alkaline conditions, the redox potential of ZnO/Zn (− 1.26 V vs. SHE) is more negative than that of the HER (− 0.83 V vs. SHE), accelerating spontaneous zinc oxidation. Continued cycling leads to irreversible zinc loss, forming “dead zinc” and insulating precipitates. The main reactions are summarized below [50]:

**Table 1:** Comparison of the performance of different zinc salts as electrolytes

Electrolytes	Cathodes	Solubility	Capacity	Cycle performance	References
1 m Zn(NO_3_)_2_	graphite paper	138 g/100 mL	Low capacity	47.4% CE at 0.02 A g^−1^	[64]
1 m Zn(CH_3_COO)_2_	graphite paper	30 g/100 mL	Low capacity	64.8% CE at 0.02 A g^−1^	[64]
0.5 m Zn(CH_3_COO)_2_	Na_3_V_2_(PO_4_)_3_	30 g/100 mL	97 mAh·g^−1^ at 0.5C	retains 74% capacity after 100 cycles	[65]
Zn(CH_3_COO)_2_	NH_4_V_4_O_10_	30 g/100 mL	281mAh g^−1^ at 500 mA g^−1^	with a capacity retention of 90.13% after 200 cycles	[66]
3 m Zn(CF_3_SO_3_)_2_	HNaV_6_O_16_·4H_2_O	Highly soluble	444mAh g^−1^at 0.5 A g^−1^	with a capacity retention ratio of 93.7% after 1000 cycles (5 A g^−1^)	[67]
3 m Zn(CF_3_SO_3_)_2_	(NH_4_)_x_V_2_O_5_·nH_2_O	Highly soluble	344 mAh g^−1^ at 0.2 A g^−1^	with 80% retention after 2,000 cycles at 0.2 A g^−1^	[68]
3 m Zn(CF_3_SO_3_)_2_	NH_4_-V_2_CT_x_/ZnO	Highly soluble	173 mAh g^−1^ at 2 A g^−1^	the average CE is almost 100% during 300 cycles at 2 A g^−1^	[69]
3 m Zn(CF_3_SO_3_)_2_	V_2_O_5_·2.2H_2_O	Soluble in water	high initial capacity (4.5 mA h cm^−2^)	25,000 cycles at 1 A g^−1^	[70]
Zn(CF_3_SO_3_)_2_	Cu	Soluble in water	High capacity	CE of 99.9% at 1 mA cm^−2^ and 1 mA h cm^−2^	[71]
3 m Zn(CF_3_SO_3_)_2_	NH_4_V_4_O_10_	Soluble in water	/	high capacity retention rate of 71.1% after 1000 cycles	[72]
1 m ZnCl_2_	Ca_0.20_V_2_O_5_∙0.80H_2_O	395 g/100 mL	296 mAh g^−1^ at 50 mA g^−1^	≈50% CE at 1.6 A g^−1^	[73]
30 m ZnCl_2_	Ca_0.20_V_2_O_5_∙0.80H_2_O	395 g/100 mL	496 mAh g^−1^ at 50 mA g^−1^	99.6% CE at 1.6 A g^−1^	[73]
3 m ZnCl_2_	NaV_3_O_8_	395 g/100 mL	362 mAh g^−1^ at 0.2 A g^−1^	84 cycles at 1 A g^−1^	[74]
3 m Zn(ClO_4_)_2_	NaV_3_O_8_	4.30 mol/kg	352 mAh g^−1^ at 0.2 A g^−1^	30 cycles at 1 A g^−1^	[74]
1 m Zn(ClO_4_)_2_	VO_2_	4.30 mol/kg	240 mAh g^−1^ at 0.5 A g^−1^	high stability for over 3500 h	[46]
3 m Zn(ClO_4_)_2_	Polyaniline	4.30 mol/kg	174 mAh g^−1^ at 0.5 A g^−1^	70,000 cycles at − 35 °C under 15 A g^−1^	[75]
1 m ZnSO_4_	Zn	53.8 g/100 mL	/	98.0% CE at 2 mA cm^−2^	[46]
3 m ZnSO_4_	V_6_O_13_ with high-content V^5+^	53.8 g/100 mL	520 mAh g^−1^ at 0.5A g^−1^	with 85.3% capacity retention at the 1000th cycle at 2 mA cm^−2^	[76]
3 m Zn(BF_4_)_2_	polyaniline and carbon nanotubes	Highly soluble	109 mAh g^−1^ at 0.1 A g^−1^	high capacity retention of 94% after 1000 cycles at 1A cm^−2^	[77]
3 m Zn(BF_4_)_2_	NaV_3_O_8_	Highly soluble	193 mAh g^−1^ at 0.2 A g^−1^	56 cycles at 1 A g^−1^	[74]

Anode:

4 $${\mathrm{Zn}}^{{{2} + }} + {\text{ 2OH}}^{ - } = {\mathrm{Zn}}\left( {{\mathrm{OH}}} \right)_{{2}} + {\text{ 2e}}^{ - }$$

5 $${\mathrm{Zn}}\left( {{\mathrm{OH}}} \right)_{{2}} = {\text{ ZnO }} + {\text{ H}}_{{2}} {\mathrm{O}}$$

Cathode:

6 $${\text{MnOOH }} + {\text{ H}}_{{2}} {\text{O }} + {\text{ e}}^{ - } = {\text{ Mn}}\left( {{\mathrm{OH}}} \right)_{{2}} + {\text{ OH}}^{ - }$$

Accumulation of byproducts, such as Zn(OH)_2_, ZnO, and Mn(OH)_2_, significantly reduces capacity and coulombic efficiency, resembling a “primary battery” [51]. Moreover, in high-concentration 6 M KOH electrolyte, the battery experienced rapid polarization within six cycles and short-circuited in 5.3 h due to aggressive dendrite formation [52]. These findings highlight the importance of electrolyte pH on battery stability.

In neutral and mildly acidic electrolytes, strong Coulombic interactions between the solvated Zn^2+^ ion and its surrounding H_2_O shell accelerate parasitic water reduction; meanwhile, the HER kinetics become highly pH-dependent. HER dominates in acidic media (2H^+^  + 2e⁻ → H_2_), competing with Zn^2+^ deposition. H_3_O⁺ ions first absorb and accept electrons on the anode surface (Volmer step), forming H* intermediates. These either recombine to form H_2_ (Tafel step) or react with more H_3_O⁺ and electrons (Heyrovsky step). In neutral conditions, the HER mechanism is more complex and influenced by multiple factors, including electrolyte concentration and species identity [53]. Zinc possesses a relatively high hydrogen evolution overpotential, which theoretically suppresses HER. This hydrogen evolution overpotential (*η*) can be expressed using the Tafel Equation:

7 $$\eta =a+b\mathrm{log}i$$

where *i* is the current density, and *a* and *b* are constants ( *b* is the Tafel slope). The Tafel slope is often used to identify the rate-determining step of the HER. Despite a high value of *a*, HER still occurs spontaneously due to practical kinetic factors, including electrode roughness, temperature, and electrolyte composition [54]. During charging, HER continuously consumes both electrons and electrolyte, reducing coulombic efficiency and shortening battery life. Accumulated H_2_ also elevates internal pressure, thereby causing battery swelling and potentially even explosion [55, 56].

**Fig. 3:**
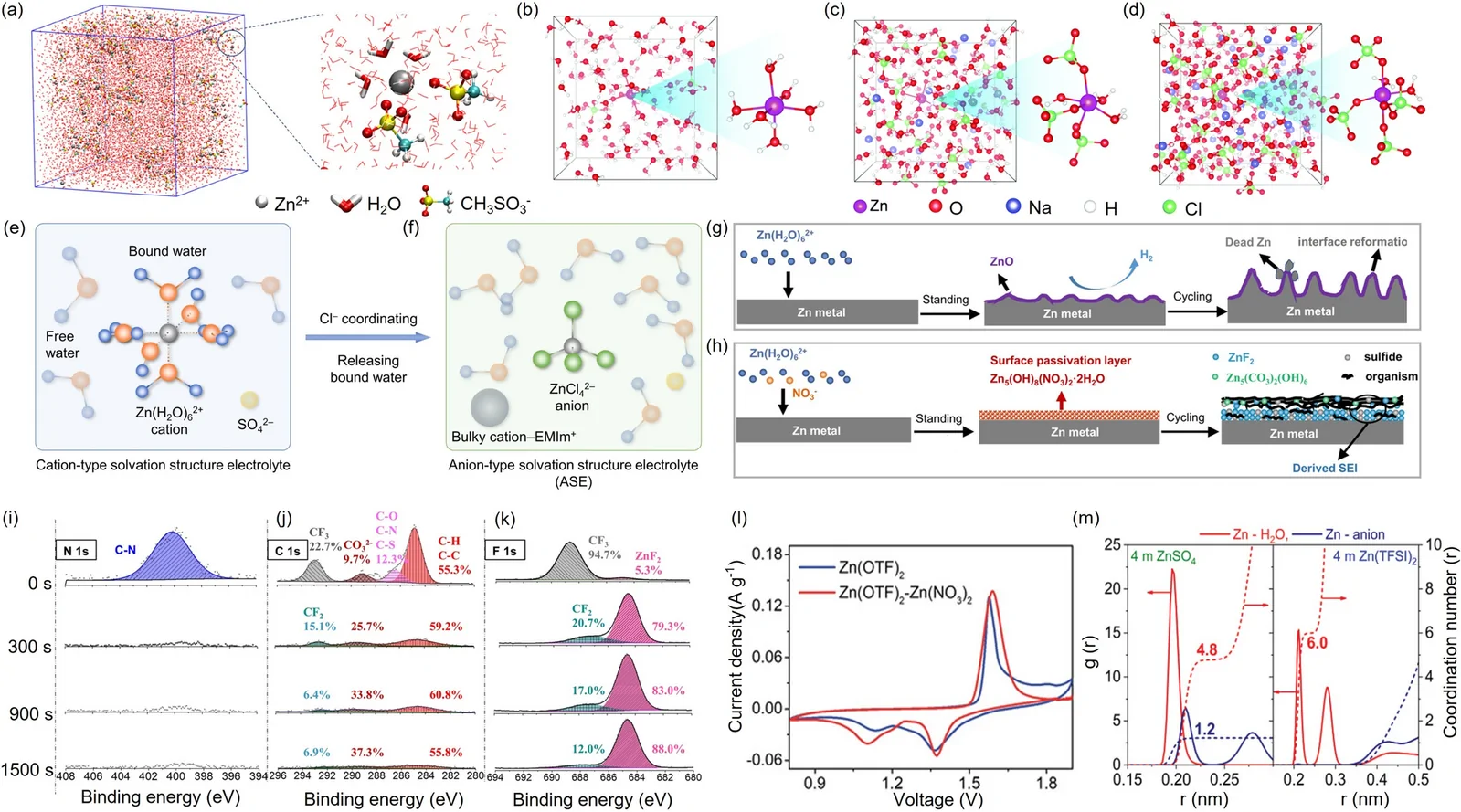
**a** Three-dimensional molecular dynamics snapshots of the Zn(CH_3_SO_3_)_2_ system, with partially enlarged view illustrating the solvation structure of Zn^2+^ [49]. Equilibrium trajectories snapshots from ab initio MD simulations of **b** 0.5 mol kg^−1^ Zn(ClO_4_)_2_, **c** 0.5 mol kg^−1^ Zn(ClO_4_)_2_ + 9 mol kg^−1^ NaClO_4_, and **d** 0.5 mol kg^−1^ Zn(ClO_4_)_2_ + 18 mol kg^−1^ NaClO_4_ [48]. **e** Representative solvation structure of Zn^2+^ ions. **f** Schematic showing how the introduction of Cl^−^ ions releases coordinated water molecules, resulting in a modified solvation structure [47]. **g** A schematic illustration of the growth of Zn dendrites in aqueous electrolytes. **h** Proposed formation mechanism of ZnF_2_-Zn_5_(CO_3_)_2_(OH)_6_-organic SEI [59]. XPS of **i** N 1* s*, **j** C 1* s*, and **k** F 1* s* with different Ar sputtering durations [59]. **l** CV of Zn||MnO_2_ full cells at a scan rate of 0.1 mV s^−1^ [59]. **m** The radial distribution function and coordination number plots of Zn^2+^solvation structure in ZnSO_4_ and Zn(TFSI)_2_ electrolyte [63]

To mitigate these issues, near-neutral zinc salts are preferred for stable cycling. Zn(CF_3_SO_3_)_2_ has gained popularity due to its effective desolvation capability (Fig. 3a) [49]. Nevertheless, other near-neutral zinc salts like Zn(NO_3_)_2_ and Zn(ClO_4_)_2_ also pose challenges. Their strong oxidizing anions can induce ZnO passivation on the anode, raising Zn^2+^ dissolution/deposition impedance and slowing kinetics. Interestingly, in a Zn(ClO_4_)_2_ and NaClO_4_ mixed system, high ClO_4_^–^ concentrations replace water in the solvation shell, reducing free water molecules and thus suppressing HER, while also modifying ion transport properties (Fig. 3b–d) [48]. Though Cl^−^ species are less oxidizing and can coordinate with Zn^2+^ to form ZnCl_4_^2−^ complexes (Fig. 3e, f) [47], AZIBs with ZnCl_2_ still suffer from poor cycling due to a limited electrochemical stability window [43]. In contrast, Zn(CF_3_SO_3_)_2_, and Zn(TFSI)_2_ are considered promising candidates owing to their wide electrochemical windows, high compatibility with electrode materials, and their anions that promote beneficial physicochemical properties like reduced solvation and suppressed water activity [57, 58].

**Fig. 4:**
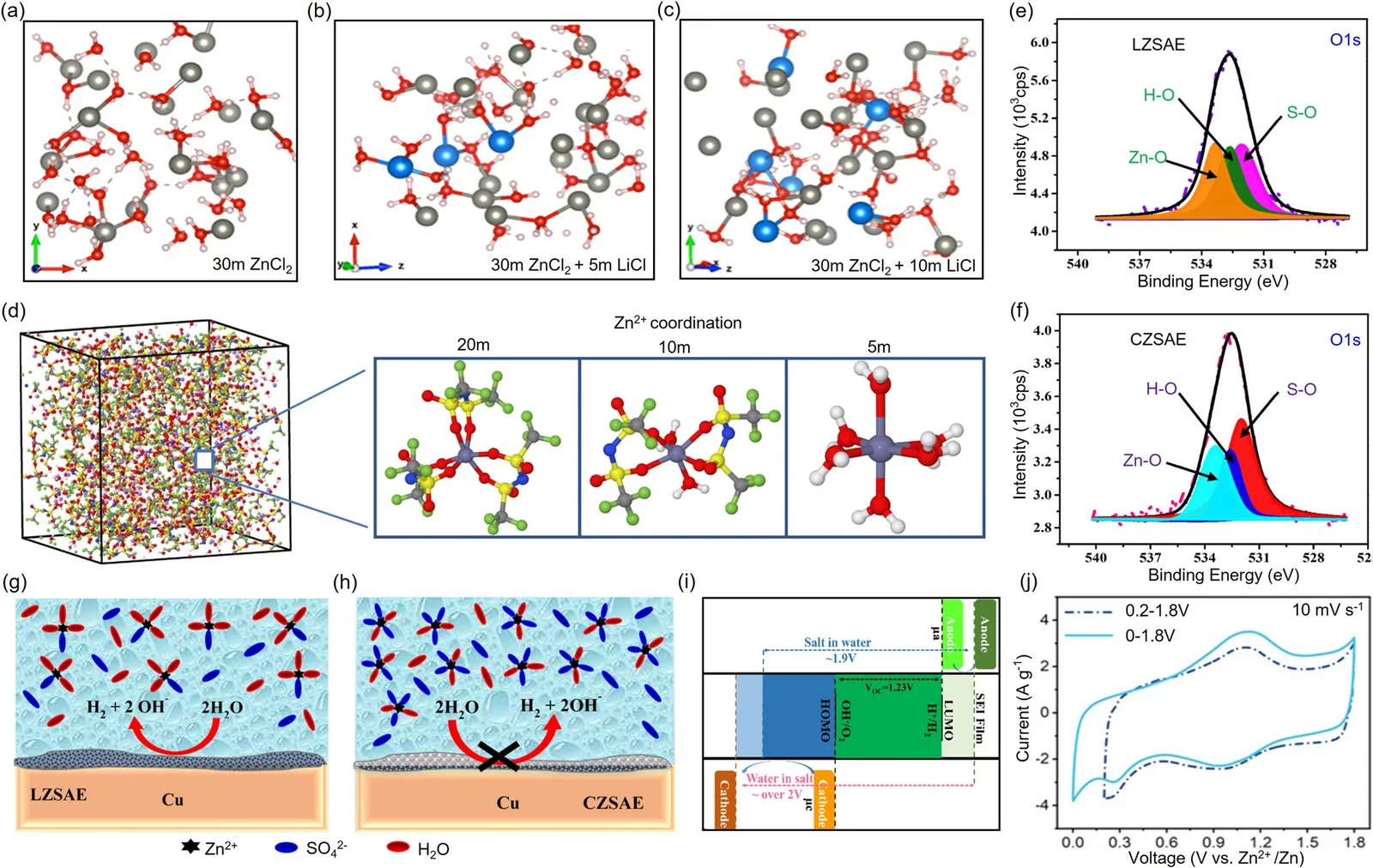
Molecular dynamics snapshots of electrolytes at various salt concentrations: **a** 30 mol kg^–1^ ZnCl_2_, **b** 30 mol kg^–1^ ZnCl_2_ + 5 mol kg^–1^ LiCl, **c** 30 mol kg^–1^ ZnCl_2_ + 10 mol kg^–1^ LiCl [78]. **d** Zn^2+^ solvation structures in electrolytes containing 1 mol kg^–1^ Zn(TFSI)_2_ with varying concentrations of LiTFSI (5, 10, and 20 mol kg^–1^) [52]. XPS investigation of O 1 s spectra showing the passivation layer formation on Cu current collectors after Zn deposition at the fifth cycle using **e** LZSAE and **f** CZSAE electrolytes [81]. Schematic illustrations of the passivation layer formation and solvation structure during Zn plating on the Cu substrate using **g** LZSAE and **h** CZSAE [81]. **i** Schematic diagram of the electrochemical stability window [79]. **j** CV curves of 7.5 m ZnCl_2_ + PAM HEs solution under two different voltage windows [83]

For instance, Li et al. introduced trace amounts of NO_3_^−^ into the Zn(CF_3_SO_3_)_2_ electrolyte, which facilitates salt decomposition and promotes the formation of a compact SEI (Fig. 3g, h). Ar sputtering-assisted X-ray photoelectron spectroscopy (XPS) analysis revealed that the SEI comprises an organic-rich outer layer and an inorganic ZnF_2_-rich inner layer (Fig. 3i–k). Additionally, cyclic voltammetry measurements demonstrated the effect of the SEI on reducing polarization voltage(Fig. 3l) [59]. In Zn(CF_3_SO_3_)_2_, the bulky CF_3_SO_3_^−^ anions effectively reduce Zn^2+^ solvation by decreasing the number of coordinated water molecules, thereby facilitating Zn^2+^ transport and improving the coulombic efficiency [60, 61]. Furthermore, CF_3_SO_3_^−^ anions can react with Zn anode to form a stable SEI, contributing to long-term cycling stability [62]. Similarly, Zn(TFSI)_2_, a commonly studied zinc salt, features a large anionic structure that disrupts strong hydrogen bonding and reduces the population of free water molecules in the electrolyte (Fig. 3m). While TFSI^−^ ions effectively suppress aqueous side reactions and promote Zn^2+^ transport, their widespread application remains limited by the high cost of the salt [63]. Beyond the type of zinc salt, increasing the electrolyte concentration is also considered an effective strategy to mitigate side reactions by regulating the interaction between Zn^2+^ and H_2_O. Table 2 summarizes the ionic conductivity, electrochemical stability window, and cycling performance of electrolytes across various concentrations, underscoring the significance of concentration engineering. Recent studies demonstrate that elevated salt concentrations enhance electrostatic interactions between anions and cations while weakening the coordination between Zn^2+^ and H_2_O. This adjustment reshapes the solvation environment and solvent sheath surrounding Zn^2+^ in the electrolyte [78, 79]. As a result, the hydration coordination number of Zn^2+^ is reduced to below 6, thereby suppressing the HER [79, 80]. For instance, in the ZnCl_2_-LiCl system, increasing LiCl concentration reduces Zn^2+^ hydration. Molecular dynamics simulations reveal that a higher Li^+^ concentration increases the number of water molecules coordinated to Li^+^, consequently decreasing the population of free water molecules and lowering HER probability (Fig. 4a–c) [78]. Similarly, in the Zn(TFSI)_2_-LiTFSI electrolyte, increasing LiTFSI concentration alters the Zn^2+^solvation structure, with the solvation sheath being predominantly occupied by TFSI^−^ anions in highly concentrated formulations (Fig. 4d) [52]. Huang et al. compared two electrolyte systems with different concentrations: a high-concentration system (4.2 m ZnSO_4_·7H_2_O + 0.1 m MnSO_4_·H_2_O, denoted as CZSAE) and a low-concentration system (2 m ZnSO_4_·7H_2_O + 0.1 m MnSO_4_·H_2_O, denoted as LZSAE). O 1* s* XPS spectra revealed that a passivation layer enriched in S–O species dominates in CZSAE, indicating increased Zn^2+^ coordination with sulfate anions and reduced interaction with water molecules(Fig. 4e, f). This solvation adjustment directly influences the composition and morphology of the passivation layer (Fig. 4g, h) [81].

**Table 2:** The effects of different electrolyte concentrations on various battery performance

Electrolytes	ESW (V)	Ionic Conductivity	Device	Cycling Performance	References
5 m ZnCl_2_	1.6	High	Zn||Zn	CE of 73.2%	[82]
10 m ZnCl_2_	2.0	High	Zn||Zn	/	[82]
15 m ZnCl_2_	1.6	12 mS cm^−1^	Zn||K_0.486_V_2_O_5_	1400 cycles with 95.02% capacity retention	[85]
15 m ZnCl_2_ + 1 m LiCl	2.4	/	Zn||LiFePO_4_	a capacity retention of 70% after 1000 cycles at 3C	[86]
20 m ZnCl_2_	2.2	Low	Zn||Zn	/	[82]
30 m ZnCl_2_	2.3	3 mS cm^−1^	Zn||Zn	CE of 95.4%	[82]
21 m LiTFSI + 2 m Zn(OTf)_2_	− 0.102 /2.745 of HER/OER	0.24 of *t*_Zn_^2+^ value	Zn||YP-80F	cycle 5220 h at 0.5 A g^−1^	[87]
21 m LiTFSI + 3 m Zn(OTf)_2_	2.6	7 mS cm^−1^	Zn||Graphite	CE of 95% over 600 cycles at 0.2 A g^−1^	[88]
1 m Zn(OTf)_2_ + 20 m LiTFSI	2.9	1.71 mS cm^−1^	Zn||LiMn_2_O_4_	CE of 99.62% at 300 cycles	[89]
30 m KAc + 3 m LiAc + 3 mZnAc_2_	2.3	6.5 mS cm^−1^	Zn||LiFePO_4_	1000 h at 0.1 mA cm^–2^	[90]
1.5 m LiAc + 1.5 m ZnAc_2_	/	High	Zn||LiMn_2_O_4_	cannot provide sufficient cyclability	[90]
30 m KAc + 1 m ZnAc_2_	2.2	High	Zn||AC	high capacity retention over 10 000 cycles	[91]
1 m KAc + 1 m ZnAc_2_	1.6	Low	Zn||AC	rapid capacity fading occurs within 10 cycles	[91]
1 m Zn(OTf)_2_	2.3	High	Zn||ZMO/C	along with a lower CE after the 3rd cycle	[43]
3 m Zn(OTf)_2_	2.5	3.47 S cm^–1^	Zn||ZMO/C	nearly 100% CE for both anode and cathode	[43]
0.5mZn(ClO_4_)_2_ + 18 m NaClO_4_	/	98.5 mS cm^−1^	Zn||NVO	Capacity retention of 80.9% after 15 days at 0.1 A g^−1^	[48]
8 m Zn(ClO_4_)_2_	2.8	/	Zn||Graphite	cycle life of over 500 cycles at 0.1 A cm^–2^	[92]
3 m Zn(ClO_4_)_2_	2.5	4.23 mS cm^−1^	Zn||MnO_2_	cycle life of over 1000 cycles at 6 A g^−1^	[93]

Additionally, recent studies have shown that a high concentration of 30 m ZnCl_2_ effectively suppresses the formation of fully hydrated [Zn(H_2_O)_6_]^2+^ complexes and instead promotes the formation of ion pairs, such as [Zn(H_2_O)Cl_4_]^2−^ and [ZnCl_4_]^2−^, more than in 20 m ZnCl_2_. Experimental results indicate that the electrochemical stability window significantly broadens with increasing ZnCl_2_ concentration, suggesting suppression of HER and the formation of inactive by-products, such as Zn(OH)_2_ and ZnO (Fig. 4i) [82]. To address the dissolution of Zn-based ion clusters in aqueous ZnCl_2_ solutions, Wang et al. developed a water-in-salt (WIS) hydrogel electrolyte containing 7.5 m ZnCl_2_. This system not only broadened the electrochemical window but also enhanced the reversibility of the Zn metal anode (Fig. 4j) [83]. Notably, the battery retained 95.1% of its initial capacity after 100,000 charge–discharge cycles. However, elevated electrolyte concentration can introduce several drawbacks, including reduced ionic conductivity, increased viscosity, and high voltage polarization, all of which hinder battery performance in practical applications [84]. Therefore, careful optimization of the electrolyte concentrations for each zinc salt is essential to balance performance, stability, and practical applicability in AZIBs.

### Electrolyte Additives

Electrolyte additives fundamentally modify the chemical and physical environment at the anode/electrolyte interface in a “trace amounts yet highly effective” manner, playing a crucial role in interfacial stability in AZIBs [94]. To function effectively, ideal electrolyte additives must possess high water solubility, a critical prerequisite that guides the screening and rational design of additive molecules. The hydrophilic and hydrophobic balance of functional groups, along with potential non-covalent interactions, should be comprehensively considered to ensure both adequate dissolution and targeted interaction at the anode/electrolyte interface. Moreover, strategies, such as pH adjustment and the use of solubilizing agents, are widely employed to enhance additive water solubility [95, 96]. Electrolyte additives must also demonstrate chemical compatibility with other battery components and avoid inducing harmful side reactions. From a practical standpoint, additives should be low-cost and exhibit minimal toxicity to meet scalability and safety requirements [97].

Based on these design principles and their mechanisms of action, commonly employed electrolyte additives for AZIBs can be broadly categorized into five groups: (i) self-healing via electrostatic shielding, (ii) surface adsorption, (iii) desolvation regulation, (iv) in situ SEI formation layer, and (v) crystal plane regulation. A detailed discussion of these mechanisms and representative examples will be presented below.

#### Self-Healing Electrostatic Shield Mechanism

The self-healing electrostatic shielding mechanism was initially proposed to enhance the performance of lithium-ion batteries. Zhang and co-workers observed that alkali-metal ions, such as Cs⁺ and Rb⁺, preferentially adsorb onto lithium-dendrite tips, forming a positively charged protective layer that redirects Li⁺ deposition toward the surrounding surface. This electrostatic shield repels incoming Li⁺ from the protrusion tip, forcing uniform deposition in adjacent regions and thereby “self-healing” nascent defects, thereby suppressing dendrite growth; this process is termed the self-healing electrostatic shield mechanism [98]. Theoretical calculations further indicate that these ions possess lower adsorption energies and diffusion barriers on lithium metal, facilitating their migration and selective accumulation at dendritic tips. In addition, they also show a tendency to prioritize the repair of SEI defects in battery systems [99].

**Fig. 5:**
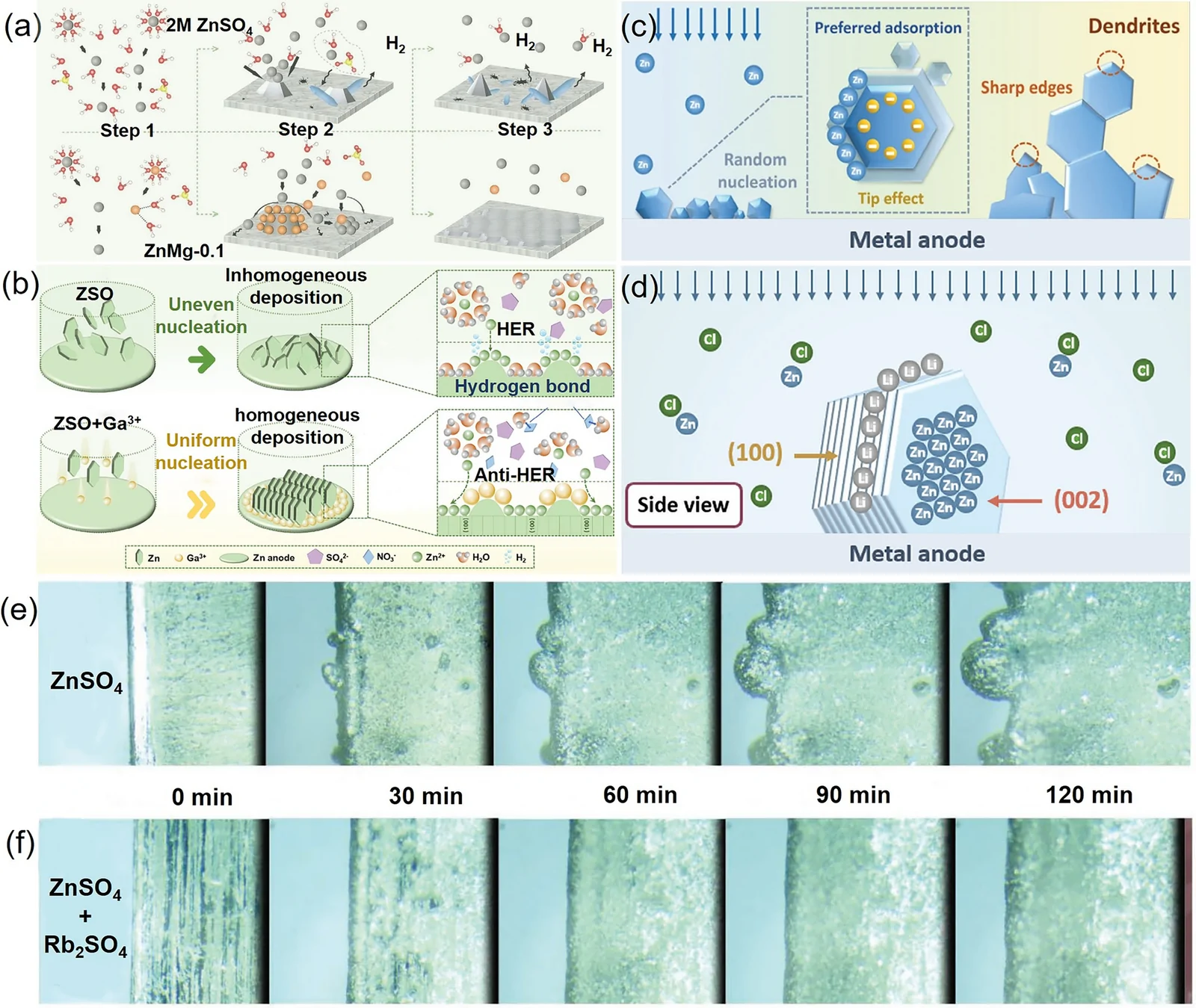
**a** Schematics illustrations of Zn deposition process in 2 M ZnSO_4_(top row) and ZnMg-0.1 (bottom row) electrolyte [103]. **b** Diagram depicting the effect of Ga^3+^ ion additives on Zn deposition process [104]. **c** Illustration of the tip effect, wherein Zn^2+^ ions preferentially adsorb to the tips and edges of the existing Zn deposit [100]. **d** Schematic showing that lithium ions preferentially adsorb on the Zn (100) plane, guiding Zn^2+^ deposition toward the (002) plane and suppressing dendritic growth [100]. In situ optical microscopic images of Zn^2+^ deposition on Zn foils at a current density of 20 mA cm^−2^ in **e** ZnSO_4_ (2 M) and **f** ZnSO_4_ (2 M) + 1.5 mM Rb_2_SO_4_ electrolytes [105]

Inspired by these findings, the strategy has been extended to AZIBs, yielding promising results. Monovalent and multivalent cations, such as Li^+^ [100], Na^+^ [101, 102], Mg^2+^ [103], Ga^3+^ [104], Rb^+^ [105], and plasma-derived additives, have been reported to form positively charged electrostatic shields around zinc deposition tips, effectively suppressing dendritic growth. For example, Yuan et al. achieved a high coulombic efficiency in Zn||Zn symmetric cells by introducing lithium halides into the electrolyte [100]. DFT calculations indicated that the adsorption energy of Zn^2+^ ion the surface of Zn (100) surface is lower than that on the Zn (200) surface [106], causing Zn^2+^ to preferentially adsorb onto the (100) face and form daisy-chain dendrites (Fig. 5c). Experimental results demonstrated that Li^+^ ions were found to preferentially adsorbed onto the (100) face due to their low binding energy, redirecting zinc nucleation toward the (002) plane and resulting in a denser, smoother anode surface (Fig. 5d).

Distinct from the Li^+^, Na^+^, and K^+^ ions, Rb^+^, with a larger ionic radius and stronger electrostatic repulsion, more effectively occupies surface protrusions, forming a broader electrostatic shield and promoting lateral Zn^2+^ deposition across the Zn surface [105]. DFT results show that Rb^+^ exhibits a significantly higher adsorption energy on metallic Zn (− 32.60 kcal mol^−1^) compared to H_2_O molecules (− 3.55 kcal mol^−1^), confirming its preferential adsorption. In addition, electrolytes containing Rb^+^ demonstrated a lower EDL capacitance(159.45 μF cm^−2^) relative to pristine 2 M ZnSO_4_ (216.09 μF cm^−2^), attributed to Rb_2_SO_4_ occupying active tip sites and altering Zn^2+^adsorption behavior (Fig. 5e, f). Thus, the Zn||Zn symmetric cells with 1.5 mmol Rb_2_SO_4_ exhibited a prolonged cycle life exceeding 6000 h at a current density of 0.5 mA cm⁻^2^ and a capacity density of 0.25 mAh cm^−2^, superior to the control group without Rb_2_SO_4_ (which cycled for 300 h).

Additionally, Wu et al. introduced a cost-effective gelatin molecule as an additive in ZnSO_4_ electrolyte [49], in which the self-healing electrostatic shield mechanism was also achieved. Owing to their steric hindrance, gelatin molecules preferentially adsorbed on the Zn surface during the electrochemical cycling. The strong Zn^2+^ binding capacity of gelatine, combined with positively charged − CN_3_H_5_^+^ groups generated under mildly acidic conditions, the 2D Zn^2+^ diffusion was effectively inhibited. This facilitates uniform zinc deposition and enhanced interfacial stability, thereby improving overall battery performance.

#### Adsorption Effects

In AZIBs, the adsorption effect of additives is considered an effective strategy to reduce the two-dimensional diffusion time of Zn^2+^ ions and lower the nucleation overpotential, thereby inhibiting the dendrite growth and mitigating side reactions. Additives with strong adsorption capabilities can form a uniformly distributed protective layer on the Zn metal surface, preventing direct contact between the anode and electrolyte. This physical barrier significantly suppresses parasitic HER and promotes uniform Zn deposition during electroplating [107]. In contrast to the self-healing electrostatic shield mechanism, which involves a repulsive layer created by cations with the same charge as Zn^2+^, neutral or even negatively charged species can also induce an adsorption effect. These species increase the density of nucleation sites and reduce the energy barrier for Zn^2+^ nucleation, thereby promoting homogeneous metal deposition. Notably, certain additives can guide Zn^2+^ deposition along a preferred crystal orientation, optimizing the microstructure of the zinc layer and enhancing electrochemical performance.

**Fig. 6:**
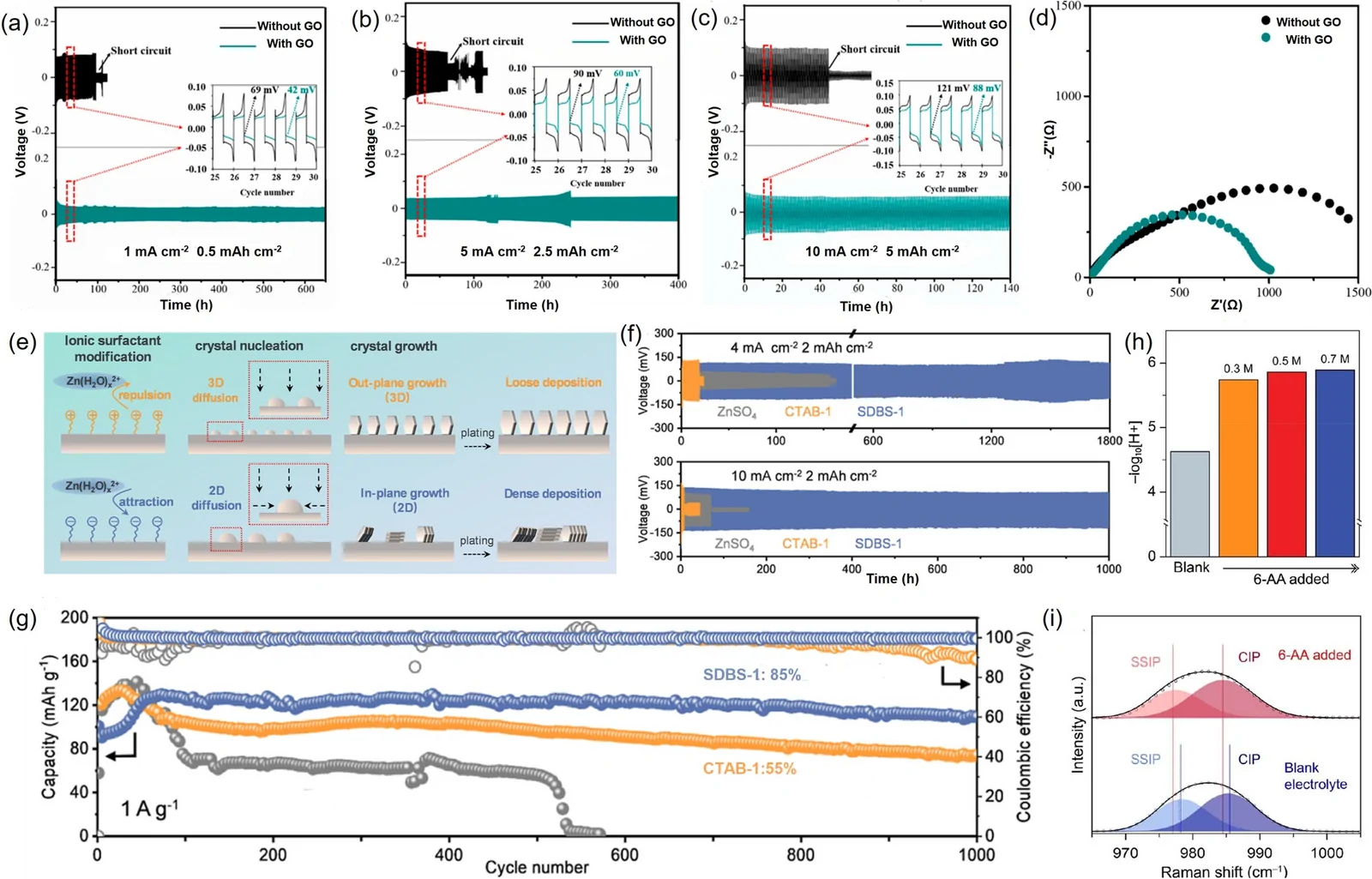
Electrochemical investigation of Zn anodes employing GO electrolyte additive [108] at **a** 1 mA cm^−2^ of 0.5 mAh cm^−2^, **b** 5 mA cm^−2^ of 2.5 mAh cm^−2^, **c** 10 mA cm^−2^ of 5 mAh cm^−2^. **d** EIS analysis of the Zn anodes with and without GO additives. **e** Schematic illustration of surfactant-modulated interactions between Zn anodes and Zn^2+^ ions, accompanied by the corresponding plating morphologies [109]. **f** Long-term cycling stability of Zn||Zn symmetric cells with a depth of 2 mAh cm^−2^ at 4 and 10 mA cm^−2^ in various surfactant-modulated electrolytes [109]. **g** Cycling performance of Zn||MnO_2_ batteries in various surfactant-modulated electrolytes at a current density of 1 A g^−1^ [109]. **h** Raman spectra of the electrolyte with 6-AA in the Raman shift range of 965–1005 cm^−1^ [113]. **i** pH values of the pristine electrolyte and the various concentrations of the 6-AA added electrolyte [113]

Among the reported additives, graphene-based materials have emerged as a particularly promising material. Qiu et al. [108] incorporated graphene oxide (GO) into the electrolyte and demonstrated that GO particles strongly adsorb onto the Zn surface through electrostatic interactions. This adsorption suppresses local electric field inhomogeneities and facilitates a more uniform field distribution, thereby lowering the Zn^2+^ nucleation overpotential. Furthermore, the abundant polar functional groups on GO surfaces accelerate Zn^2+^ migration to the reaction interface, increasing active nucleation sites and enhancing ion diffusion, resulting in a denser and smoother zinc deposit. As a result, the AZIBs using GO additive exhibited remarkable stability, operating continuously for over 650 h at a current density of 1 mA cm^−2^ (Fig. 6a), and maintaining stable cycling for 400 and 140 h at 5 mA cm^−2^ (Fig. 6b) and 10 mA cm^−2^, respectively (Fig. 6c). Electrochemical impedance spectroscopy (EIS) analysis revealed significantly reduced resistance in GO-containing systems (Fig. 6d), indicating improved charge transfer kinetics. Additionally, the system achieved a high coulombic efficiency of 99.16%, which is superior to the conventional ZnSO_4_ electrolyte in both cycling stability and energy conversion efficiency.

Building upon insights into the rate of change of electrochemical surface area (dS/dt), Xie et al. developed two nucleation models, the instantaneous nucleation model (INM) and the continuous nucleation model (CNM). The INM describes limited two-dimensional diffusion under conditions of weak electrostatic adsorption of Zn^2+^, while CNM occurs when Zn^2+^ adsorption is enhanced, facilitating continuous two-dimensional diffusion and nucleation (Fig. 6e) [109]. The authors demonstrated that ZnSO_4_ electrolytes, due to the relatively weak electrostatic adsorption with Zn^2+^, favor the INM. In this scenario, multiple independent nuclei form rapidly at the onset of electrodeposition and subsequently grow via three-dimensional diffusion. However, this leads to a loosely packed zinc layer prone to dendrite formation, as evidenced by prominent dendrite growth when cetyltrimethylammonium bromide (CTAB) was used as an additive. In contrast, the introduction of sodium dodecylbenzene sulfonate (SDBS) into the electrolyte led to continuous nucleation, with new crystal nuclei forming both during the early and later stages of deposition (Fig. 6f, g). This behavior indicates enhanced Zn^2+^ electro-adsorption and supports a CNM mechanism, which promotes denser and more planar Zn deposition through a dominant two-dimensional diffusion pathway. This continuous nucleation mechanism has also proven effective for other long-chain anionic surfactants, such as the anionic part of sodium dodecyl sulfate (SDS) [110], and provides a promising strategy for the rational design of Zn anodes [111].

Remarkable adsorption properties were also observed in electrolytes containing ethylenediaminetetraacetic acid (EDTA) anions. DFT calculations indicated the adsorption energy of EDTA anions on the Zn surface is − 1.62 eV, significantly lower than that of free H_2_O (− 0.31 eV) and the Zn(H_2_O)_6_^2+^ complex (− 0.12 eV), indicating a stronger affinity of the Zn surface for EDTA anions. Owing to their firm adsorption, EDTA anions effectively block HER sites on Zn surface, thereby suppressing parasitic HER during both plating and stripping processes. Furthermore, the strong complexation between EDTA and Zn^2+^ provides additional nucleation sites during the initial deposition stage, which effectively facilitates grain refinement. The adsorption layer formed by EDTA also facilitates the formation of nanoscale ion-transport pathways, preventing direct water access and mitigating H_2_O interference in the deposition process. These combined effects contribute to uniform and flat zinc deposition and enhance the cycling stability. Experimental results indicated that a symmetric cell with tetrasodium EDTA achieves over 2000 h of stable cycling at current densities of 5 mA cm^−2^, approximately 30 times longer than the pristine ZnSO_4_ electrolyte [111]. Besides, Wang et al. demonstrated that 0.04 M EDTA, when added to a zinc-vanadium pentoxide battery system, improved the coulombic efficiency to 99.36% and enabled over 4000 h of stable operation, confirming EDTA’s effectiveness in boosting AZIBs’ performance [112]..

Similarly, the zwitterionic molecule 6-aminocaproic acid (6-AA) has shown potential to enhance battery performance through surface adsorption effects [113]. As illustrated by the Raman shift in Fig. 6i, the cationic portion (amino group) of 6-AA can interact with Zn^2+^ ions on the Zn metal surface via physical adsorption or chemical bonding, creating a barrier that inhibits water molecules and other potential reactive ions’ access and reduces side reactions.

#### Desolvation

During the electrodeposition of Zn^2+^, the desolvation of hydrated zinc ions of [Zn(H_2_O)_6_]^2+^ is a critical step that significantly affects Zn deposition behavior. To enable long-term stable performance of AZIBs, this process, requiring partial or complete removal of water molecules from the solvation shell, must effectively reverse the initial solvation of Zn^2+^ [114, 115]. Notably, the water molecules released during the desolvation process are often more reactive than free water [116], which increases the energy barrier for desolvation, and subsequently hinders Zn^2+^ diffusion and deposition kinetics [117]. In addition to slowing ion transport, these highly reactive water molecules could attack the cathode lattice structure, leading to cathode dissolution, irreversible phase transitions, and overall degradation of electrochemical stability. Thus, regulating the desolvation behavior of [Zn(H_2_O)_6_]^2+^ by rational electrolyte design is essential to achieve uniform and efficient zinc deposition [118, 119].

**Fig. 7:**
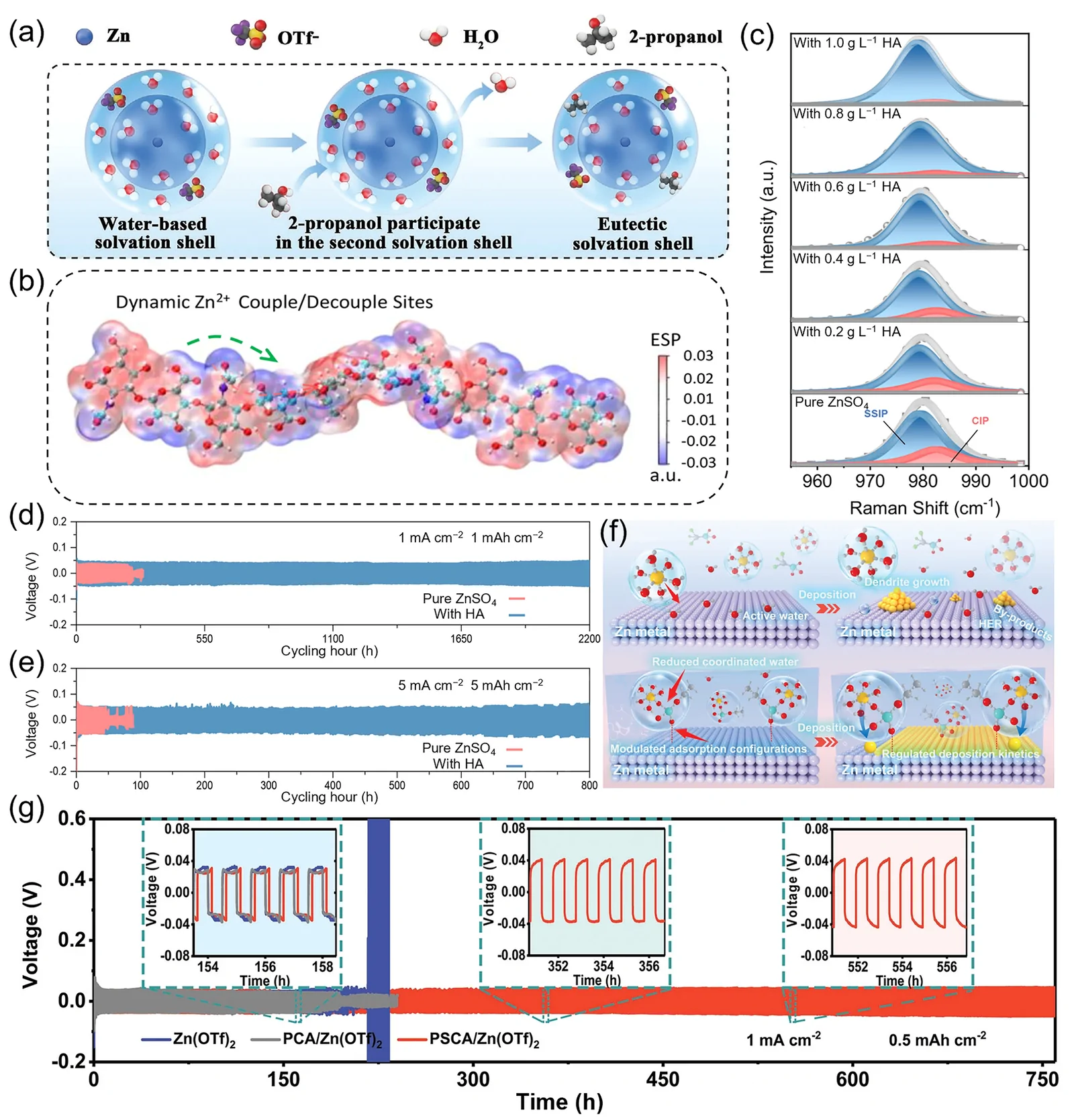
**a** Schematic showing the participation of 2-propanol in the second solvation shell of Zn^2+^ ions [120]. **b** Electrostatic potential mapping of the HA molecule. **c** Raman spectra for pristine ZnSO_4_ and ZnSO_4_ − HA with varying amounts of HA. Long-term cycling stability comparison for **d** pristine ZnSO_4_ and **e** ZnSO_4_ − HA electrolyte systems [121]. **f** Schematic illustration of the solvated Zn^2+^ deposition behavior in pristine Zn(OTf)_2_ and PSCA/Zn(OTf)_2_ hydrogel electrolyte [122]. **g** Cycling tests of the Zn||Zn cells in various electrolytes at 1 mA cm^−2^ and 0.5 mAh cm^−2^ [122]

Recent advances in electrolyte engineering have made significant progress by tailoring the Zn^2+^ solvation structure. Ma et al. focused on manipulating the outer solvation shell through 2-propanol additives, which establish a eutectic solvent network that preferentially interacts with Zn (101) crystal planes (Fig. 7a) [120]. The 2-propanol-water eutectic structure exhibits strong directional affinity toward the Zn (101), promoting oriented Zn deposition and accelerating desolvation kinetics, particularly at low temperature conditions, thus delivering fast and reliable electrochemical performance. Moreover, 2-propanol disrupts the pre-existing H_2_O − H_2_O hydrogen bonding network by forming new 2-propanol − H_2_O hydrogen bonds. Its hydroxyl groups also serve as coordination sites for Zn^2+^, contributing to the formation of efficient ion transport pathways and enhancing Zn^2+^ mobility.

Qiu et al. introduced an innovative strategy by incorporating hyaluronic acid (HA), a chaotropic polymer-type additive, into the electrolyte, which effectively suppressed undesired dendrite growth and side product formation on the Zn anode (Fig. 7b) [121]. As shown in Raman spectra (Fig. 7c), the HA additive disrupts the original hydrogen-bonded network of H_2_O−H_2_O interactions, reducing the number of active water molecules. This disruption leads to the formation of new HA−H_2_O hydrogen bonds, thereby mitigating parasitic reactions driven by highly reactive water species. Additionally, abundant functional groups, such as carboxyl (−COOH) and hydroxyl (−OH), distributed along the HA polymer chains serve as binding sites for Zn^2+^, facilitating the formation of effective ion transport channels and enhancing Zn^2+^ transfer. The dynamic coupling/decoupling behavior of Zn^2+^ along the flexible HA chains elevates the Zn^2+^ transference number to 0.62, significantly improving the cycling life of Zn||Zn symmetric cells. As a result, the constructed AZIBs achieved outstanding cycling stability, with lifetimes of 2,200 h at 1 mA cm^−2^/1 mAh cm^−2^ (Fig. 7d) and 800 h at 5 mA cm^−2^/5 mAh cm^−2^ (Fig. 7e), respectively.

To further regulate the hydration structure of Zn^2+^, a zincophilic anionic hydrogel electrolyte (PSCA/Zn(OTf)_2_) was developed, incorporating dodecyl sulfate anions ((OSO_3_R)⁻) micelles to suppress dendrite growth and parasitic reactions on the Zn anode (Fig. 7f) [122]. Experimental evidence revealed that (OSO_3_R)⁻ anions disrupt the native H_2_O − H_2_O hydrogen-bond network, reducing the population of active water molecules, and form new (OSO_3_R)⁻-H_2_O hydrogen bonds. This suppresses passivation and HER. Furthermore, functional groups along the (OSO_3_R)⁻ chains provide Zn^2+^ binding sites, enabling the construction of effective ion transport pathways and enhancing Zn^2+^ transference. This design significantly improves the lifetime of Zn||Zn symmetric cells, achieving 760 h at 1 mA cm^−2^/0.5 mAh cm^−2^. Additionally (Fig. 7g), dynamic ion-polymer interactions along flexible (OSO_3_R)⁻ chains further promote Zn^2+^ mobility, resulting in ultra-long cycling stability of over 40,000 cycles with a capacity decay of only 0.00027% per cycle.

In addition to the small-molecule additives, polymeric additives also possess unique capabilities in modulating Zn^2+^ solvation structure [95]. He et al. employed a nonionic amphiphilic polymer additive (APA) to establish a nano-scaled hydrophobic confinement layer at the anode–electrolyte interface. The hydrophilic acrylamide segments of APA preferentially adsorb onto the Zn metal surface, while the hydrophobic methacrylate segments form a water-repellent shell that locally suppresses H_2_O activity. This amphiphilic structure induces a partial desolvation of Zn^2+^ by replacing coordinated water molecules with polymer segments, thereby minimizing water-related side reactions. This strategy achieves exceptional cycling stability, exceeding 8,800 h at 1 mA cm^−2^/1 mAh cm^−2^ [123]. Similarly, Zhang et al. introduced poly(acrylic acid) (PAA) to reconstruct the Zn^2+^ solvation structure from [Zn(H_2_O)_6_]^2+^ to a more conductive [Zn(H_2_O)_2_(AA)_4_]^2−^ complex, thereby accelerating charge-transfer kinetics. The carboxyl moieties in PAA self-assemble into a hydrophobic interfacial layer that isolates the Zn anode from bulk water, promoting Zn^2+^ desolvation and uniform electrodeposition, ultimately enhancing cycling reversibility [124].

#### *Formation of *In Situ* SEI Layer*

The formation of the SEI is closely associated with the decomposition of solvents and additives in the electrolyte. Structurally, the SEI typically comprises two distinct layers: a dense inner layer and a porous outer (diffuse) layer. The dense layer is primarily composed of elements, such as Zn, F, and O, possessing high mechanical strength and compactness; whereas the outer layer mainly consists of organic compounds, offering flexibility and porosity. This dual-layer architecture effectively mitigates direct contact between the Zn anode and the electrolyte, thereby mitigating side reactions and dendrite growth. Furthermore, the intimate adhesion between the diffuse and dense layers prevents SEI cracking and facilitates Zn^2+^ ions transport. However, in aqueous electrolytes, the formation of an in situ SEI layer remains challenging due to intense HER and the difficulty in decomposing zinc salts [125]. Current efforts focus on constructing artificial SEI layers using inorganic or organic composite coatings (e.g., MOFs and COFs) or introducing trace electrolyte additives [9, 122, 126–129]. Among these approaches, electrolyte additives are particularly attractive due to their operational simplicity, low cost, and compatibility with commercial-scale applications.

**Fig. 8:**
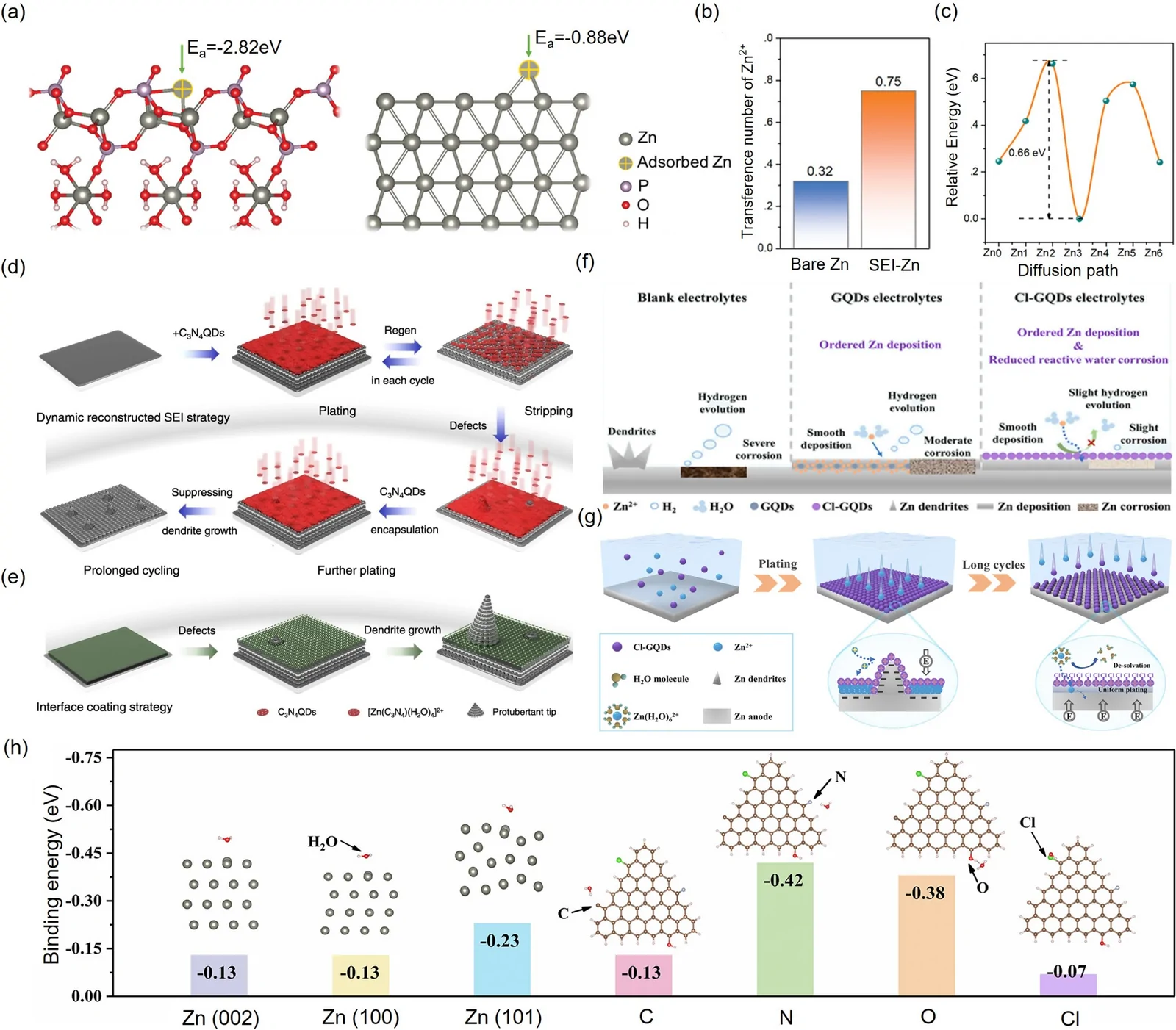
**a** Calculated interaction models of Zn^2+^ ion with the Zn_3_(PO_4_)_2_·4H_2_O SEI layer (left) and bare zinc anode surfaces (right), respectively [130]. **b** Comparison of Zn^2+^ ion transference numbers between bare Zn anode and Zn_3_(PO_4_)_2_ 4H_2_O SEI-coated Zn anode [130]. **c** Calculation of the corresponding migration energy barriers in the Zn_3_(PO_4_)_2_ 4H_2_O SEI layer [130]. **d** Schematic of real-time dynamic interphase reconstruction with C_3_N_4_QDs as the additives. **e** Failure mechanism of stationary interface protective coatings [15]. **f** Schematic diagram of Zn plating and corrosion in pristine, GQD-based and Cl-GQD-based ZnSO_4_ electrolytes [139]. **g** Schematic diagram of the morphological evolution of the Zn anode in Cl-GOD-based ZnSO_4_ electrolyte [139]. **h** Binding energies of a water molecule to the Zn (002), (100), (101) crystal plane, and different functional groups in GQDs [139]

Inspired by the phosphating solution Zn(H_2_PO_4_)_2_ in the electroplating, where a phosphide layer forms on Zn, Guo et al. introduced the Zn(H_2_PO_4_)_2_ into a ZnSO_4_ electrolyte to induce the in situ formation of an inorganic Zn_3_(PO_4_)_2_ SEI layer, significantly enhancing the reversibility of the Zn anode [130]. This dense, inorganic-rich SEI acts as an effective physical barrier, preventing water penetration and thus suppressing the HER and passivation. The OH⁻-mediated formation of the SEI endows it with strong Zn^2+^ adsorption capacity, substantially higher than that of the bare zinc anode (Fig. 8a), thereby increasing the Zn^2+^ transference number (Fig. 8b) and reducing the diffusion energy barrier (Fig. 8c). Beyond phosphate-based layers, fluorinated SEI has also been extensively studied for AZIBs. For instance, Cao et al. employed Me_3_EtNOTf as an additive in the Zn(OTf)_2_ electrolytes, leading to the in situ formation of a 64 nm ZnF_2_ layer, as confirmed by transmission electron microscopy. This layer acts as an electronic barrier to suppress water reduction, while permitting Zn^2+^ transport [131]. Luo et al. constructed a multifunctional in situ SEI composed of PNM-ZnF_2_-ZnS-ZnSO_x_ that blocks water and anion penetration, while enabling selective Zn^2+^ shuttling [132]. Other strategies have also proven effective [133–136]. Wu et al. constructed a dense SEI layer through chelation-induced polymerization of Zn^2+^ with poly(N-[2-(3,4-dihydroxyphenyl)ethyl]-2-methylacrylamide) [137]. Huang et al. introduced saccharin (Sac) as a zincophilic additive. The Sac anion strongly adsorbs onto the Zn surface, forming an electric double layer that repels H_2_O and regulates Zn^2+^ diffusion, thereby suppressing dendrite growth and limiting the water access essential for HER and subsequent passivation [138].

However, the in situ formation of the SEI is inherently uncontrollable, making it difficult to precisely regulate its structure, composition, thickness, and compactness. Moreover, current strategies for constructing interfacial layers often suffer from limitations during battery cycling, such as layer fracture, inability to regenerate, or the continuous consumption of electrolyte and functional components. Given that the deposition/stripping of Zn^2+^ ions at the reaction interface is a dynamic process, relying on static interfacial modifications is insufficient to accommodate long-term changes in interfacial microstructure and local electrochemical microenvironments. Therefore, establishing a dynamically stable and compact interface that enables real-time, directional control of interfacial reactions is essential. To address this, our team employed graphitic carbon nitride quantum dots (C_3_N_4_QDs) as an example to evidence this concept of constructing a dynamic self-repairing protective interface [15]. These C_3_N_4_QDs act as highly efficient colloidal ion carriers in the electrolyte, exhibiting strong interactions with Zn^2+^, thereby optimizing their solvation structure and suppressing water activity. Additionally, C_3_N_4_QDs self-assemble horizontally on the Zn anode to form a protective SEI layer. Their periodic sub-nanometer pores are oriented perpendicular to the electrode surface, forming directional ion channels that selectively allow the transport of Zn^2+^ ions while excluding solvated species, effectively achieving a molecular sieving effect. Upon electric field reversal, the C_3_N_4_QDs that were previously tightly attached to the surface of the Zn anode can desorb and return to the bulk electrolyte via coulombic interactions (Fig. 8d, e). This dynamic regeneration occurs repeatedly during each charge–discharge cycle. Consequently, defects or early stage dendrite formation can be repaired in subsequent cycles through C_3_N_4_QDs redistribution. This mechanism actively corrects Zn deposition behavior, fundamentally eliminating the irreversible interface rupture and maintaining long-term interfacial stability. Similarly, Wang et al. introduced positively charged chlorinated graphene quantum dots (Cl-GQDs) as additives. During charging, Cl-GQDs electrostatically adsorb onto the Zn surface, forming a shielding layer. Their chlorine groups exhibit hydrophobic characteristics that further promote uniform Zn deposition (Fig. 8f, g). Upon discharge, Cl-GQDs undergo dynamic regeneration and redissolve into the electrolyte, ensuring a continuous and stable interfacial layer [139]. Theoretical calculations and electrochemical characterizations revealed that Cl-functionalized GQDs exhibit significantly lower binding energies with water molecules (Fig. 8h), confirming their superior hydrophobicity and suitability for constructing robust interfacial layers. Notably, this dynamic self-healing process does not consume the additives, enabling sustained interfacial protection and contributing to significantly enhanced performance of AZIBs.

#### Regulation of the Zinc Crystal Plane

**Fig. 9:**
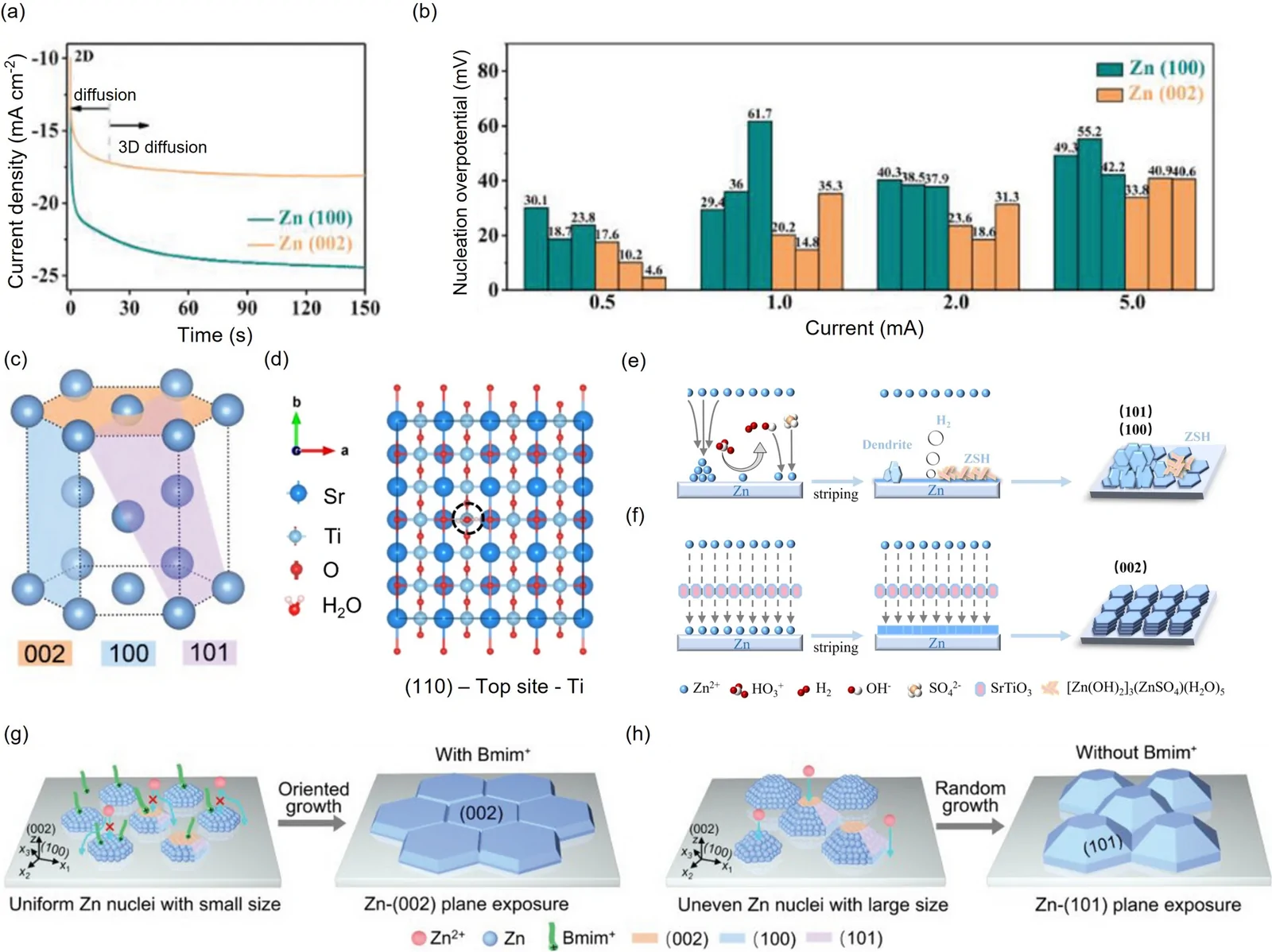
**a** Chronoamperograms of Zn anodes under constant potential conditions [143]. **b** Comparison of nucleation overpotentials of Zn (100) and Zn (002) planes, derived from three independent symmetric cell experiments at varying current densities [143]. **c** Illustration of the hexagonal close-packed crystal structure of Zn [154]. **d** Top view of the geometrical configurations of H_2_O adsorbed on the Ti atom of SrTiO_3_ (110) plane [150]. Schematic diagram of the zinc deposition processes in **e** the conventional electrolyte and **f** the densified electrolyte [150]. Schematic illustration of Zn electrodeposition in **g** Bmim-containing and **h** Bmim-free electrolytes [154]

Understanding the crystallographic behavior of the Zn anode, particularly the distinct electrochemical properties of its crystal facets, is essential for designing and evaluating interfacial strategies in electrolyte engineering. Electrolyte components can markedly influence Zn nucleation and growth orientation via preferential adsorption, thereby dictating deposition morphology and long-term stability [140, 141]. A central goal in optimizing Zn-deposition morphology is to promote preferential growth along the (002) crystal plane. This facet orientation plays a critical role in determining corrosion resistance and the dendrite growth direction of the zinc anode. Crystallographically, during the initial nucleation stage, Zn atoms tend to nucleate on low-energy planes such as Zn (101) and Zn (100) due to the polycrystalline nature of zinc and the anisotropy inherent in its hexagonal close-packed (hcp) lattice. As deposition proceeds, Zn atoms commonly adopt a two-dimensional hexagonal morphology with sharp edges and continue to grow along these crystal planes [142]. In general, a larger angle between the growth direction of Zn dendrites and the anode surface correlates with increased dendrite formation. Under constant voltage, the current response for Zn (002) remains stable after an initial brief two-dimensional diffusion phase, indicative of a steady three-dimensional diffusion process (Fig. 9a). This behavior suggests that the Zn (002) crystal plane exhibits a lower nucleation overpotential and reduced energy barrier for critical nucleus formation (Fig. 9b), making it an ideal target for electrolyte additives aiming to induce uniform nucleation and suppress dendrite formation. The planar symmetric structure and close-packed nature of the (002) crystal plane facilitate the uniform and dense deposition of zinc atoms, effectively eliminating dendrite growth (Fig. 9c). This deposition mode represents an ideal benchmark in electrolyte design. Furthermore, the (002) crystal plane exhibits relatively low HER activity, reducing the likelihood of parasitic side reactions [143, 144]. Other crystal planes, such as Zn (103) and Zn (105), also demonstrate low HER activity, owing to their small angle (less than 30°) relative to the substrate surface, further supporting their potential in dendrite suppression strategies [145].

Constructing a Zn metal anode with a dominant (002) crystal plane orientation has emerged as a simple yet effective strategy for achieving high-performance AZIBs. However, conventional approaches such as etching and epitaxial electrodeposition often involve complex procedures and limited scalability [146–148]. Recent studies have demonstrated that specific electrolyte additives can effectively induce zinc deposition with preferred orientation origination by selectively adsorbing onto specific crystal planes. Inorganic additives, such as indium sulfate, tin oxide, and boric acid [143, 149], have been shown to regulate crystal orientation and inhibit the growth of dendrites. For instance, Deng et al. developed a densified aqueous electrolyte incorporating SrTiO_3_ metal oxide as an additive to achieve high-performance AZIBs [150]. DFT calculations revealed that Ti atoms on the SrTiO_3_ (110) plane exhibit high binding energy with water molecules, indicating a strong water affinity. Consequently, SrTiO_3_ particles adsorb water molecules, altering Zn^2+^ solvation structure and facilitating the incorporation of SO_4_^2−^ into the solvation shell (Fig. 9d). Meanwhile, SrTiO_3_ exhibits excellent affinity for Zn^2+^ ions, thereby guiding zinc deposition along the Zn (002) plane (Fig. 9e, f), enabling a Frank–Van der Merwe layer-by-layer growth mechanism. As a result, Zn||MnO_2_ cells assembled with this densified electrolyte delivered a high specific capacity of 328.2 mAh g^−1^ at 1 A g^−1^ after 500 cycles.

Organic additives, such as sulfonate anions, alcohols, and sugars, have also demonstrated the ability to tailor zinc texture by interacting with specific crystallographic planes [61, 96, 151, 152]. Certain polymers, owing to their specific functional groups, can preferentially adsorb onto the anode surface and promote uniform Zn^2+^deposition along the (002) crystal plane [153]. Ma et al. proposed a novel organic cation-assisted non-epitaxial electrodeposition strategy using 1-butyl-3-methylimidazolium cation (Bmim) as a paradigm additive [154]. Mechanistic studies indicated that Bmim^+^ cations selectively adsorb on the Zn (002) plane while simultaneously suppressing the growth rate of this plane, ultimately exposing and stabilizing the (002) orientation (Fig. 9g, h). The synergistic effect of the textured (002)Zn anode and the Bmim-containing electrolyte endows excellent cycling stability for over 350 h at 20 mAh cm^−2^ with a discharge depth of 72.6%.

### New-Type of Electrolytes

In AZIBs, liquid electrolytes are widely adopted due to their high ionic conductivity. However, the application also introduces challenges, such as persistent side reactions and the instability at the solid–liquid interface, which limit further enhancements in battery performance [155, 156]. To overcome these limitations, hydrated eutectic electrolytes and gel electrolytes have emerged as promising alternatives for the next generation of AZIBs.

#### Hydrated Eutectic Electrolytes

Hydrated eutectic systems are formed by dissolving another crystallographically compatible salt or certain organic additives into hydrated salts, enabling the formation of eutectic mixtures with high salt concentrations and extended electrochemical stability windows. These systems can alter the Zn^2+^ solvation structure and reconstruct the interfacial phase of the EDL, thereby expanding the electrochemical stability window and enhancing ion transport [157, 158].

Under supersaturated conditions, ions disrupt the hydrogen-bond network of both coordinated and free water molecules, resulting in suppressed water activity, broader stability window, and improved ionic conductivity, all of which contribute to high-performance AZIBs [159, 160]. By tuning the composition, hydrated eutectic electrolytes significantly alter the solvation structure of Zn^2^⁺ and reinforce the hydrogen bonding, effectively immobilizing free water molecules. The synergistic effect between the optimized solvation and the strengthened hydrogen-bonding networks collectively imparts the electrolyte with enhanced capabilities to suppress side reactions and facilitate ion transport, thereby significantly improving the overall battery performance. Chen et al. developed a hydrated eutectic electrolyte based on ethylene glycol (EG), Zn(OTf)_2_, and a trace amount of H_2_O. By precisely controlling the water content, they minimized the number of active water molecules. In this system, EG and OTf⁻ anions cooperatively coordinate with Zn^2^⁺ ions via hydrogen bonds, establishing a thermodynamically stable solvation structure of [Zn(H_2_O)_2_(EG)_2_(OTf)_2_]. This architecture confines water activity and stabilizes the solvation sheath, resulting in a wide electrochemical stability window, low viscosity, and a high ion transference number [161]. More recently, Jiang et al. proposed a dual-salt high concentration electrolyte (15 mol kg^–1^ ZnCl_2_ + 10 mol kg^–1^ NH_4_NH_2_SO_3_), denoted as DS-HCE. The modulated solvation structure in DS-HCE delivers high ionic conductivity and ultra-low water activity. Compared to the conventional 30 M ZnCl_2_, DS-HCE achieves a broader electrochemical stability window. As a result, Zn||Zn symmetric cells using DS-HCE exhibit outstanding cycling stability, maintaining a lifespan of 2,200 h at 0.5 mA cm⁻^2^ and 0.5 mAh cm⁻^2^ [162].

#### Gel Electrolytes

**Fig. 10:**
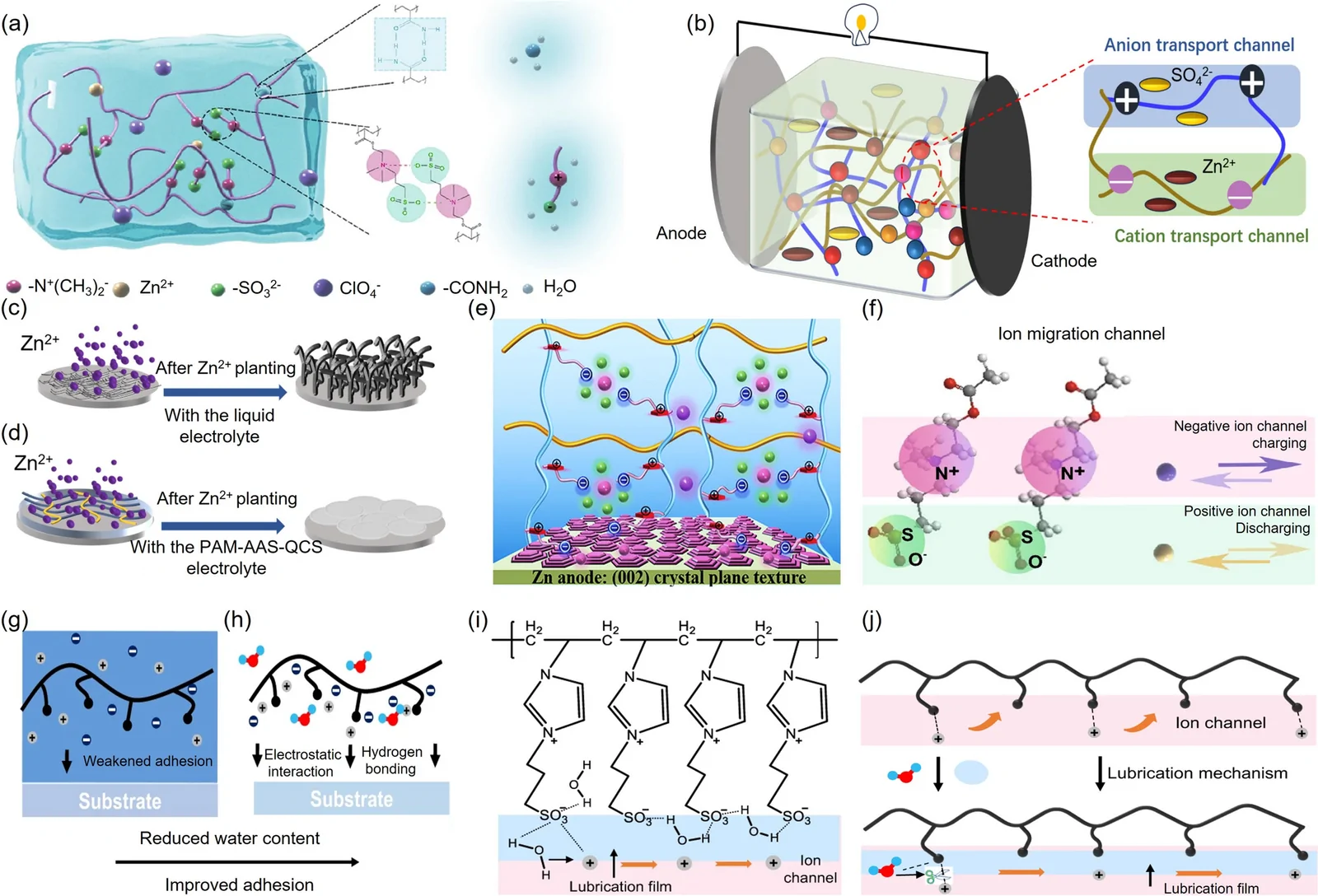
**a** Schematic illustration of intermolecular interactions within the ADC-gel matrix [171]. **b** Schematic representation of ion migration channels formed in PAM-AAS-QCS hydrogel electrolyte [166]. Schematic diagrams comparing Zn plating behaviors in **c** conventional liquid electrolyte and **d** the PAM-AAS-QCS hydrogel electrolyte [166]. **e** Mechanism of Zn anode texture regulation using gel electrolyte [167]. **f** Schematic depiction of ion channel formation under the applied electric field [171]. Illustration of the adhesive mechanism of zwitterion hydrogel electrolyte at **g** high and **h** low water contents [175]. **i** Functional mechanism of zwitterion hydrogel electrolytes [175]. **j** Ionic transportation mechanism under lean-water conditions: including the formation of directional ion transportation channels and lubrication mechanism [175]

Gel electrolytes, which combine the structural stability of solid-state electrolytes with the high ionic kinetics of liquid electrolytes, have also become a focal point of research in the field of AZIBs [163, 164]. Compared to conventional aqueous electrolytes, gel electrolytes typically feature a porous and three-dimensional network composed of functional chemical components that facilitate the creation of efficient ion transport channels and enable uniform Zn^2+^ deposition (Fig. 10a, b) [165, 166]. Recent studies have shown that polar functional groups, such as hydroxyl, carboxyl, and sulfonic groups, are widely present in gel electrolytes. These groups promote directional Zn^2+^ migration, enhance ionic conductivity, and reduce overpotential induced by concentration polarization. For instance, Zeng et al. introduced amphiphilic groups into a hydrogel electrolyte, enabling three-dimensional Zn^2+^ diffusion and guiding the formation of crystalline zinc deposits (Fig. 10c, d) [166]. Furthermore, charged functional groups (e.g., sulfonate and imidazole groups) can modulate the Zn^2+^ solvated structure and induce preferential growth along the Zn (002) crystal plane (Fig. 10e) [167]. The hydrophilic segments within gel electrolytes form hydrogen bonds with water molecules, effectively reducing the content of free water, thereby minimizing side reactions and providing a safer and more stable electrochemical environment [168–170]. Building on this strategy, He et al. incorporated a betaine-type zwitterionic monomer into Zn(ClO_4_)_2_ and copolymerized it with acrylamide to construct a gel framework with both hydrophilicity and charged groups (denoted as ADC-gel). The interaction between water and hydrophilic groups imparts high water retention, while the polymer backbone, under an applied electric field, forms directional ion migration channels along the aligned zwitterionic side chains, thereby enhancing Zn^2+^ transport efficiency (Fig. 10f). This gel electrolyte confers an extremely long cycle life on Zn||Zn symmetric batteries, with a cycle life exceeding 3,000 h at a current density of 0.5 mA cm^−2^ [171].

Despite these advancements, gel electrolytes still face challenges, including low ionic conductivity, limited mechanical strength, and poor environmental sustainability [172]. To address these issues, water-in-salt hydrogel electrolytes have been proposed [173, 174]. These systems suppress H_2_O activity, broaden the electrochemical stability window, and improve ionic conductivity [157]. Their low water content enhances interfacial adhesion, offering promise for flexible and wearable energy storage devices (Fig. 10g, h). For instance, Wang et al. reported a lean-water hydrogel electrolyte based on a polymeric zwitterion. The sulfonate groups exhibited both hydrophilic and zincophilic characteristics, facilitating Zn^2+^ transport. In this low-water environment, interactions between water molecules and charged groups result in the formation of molecular lubrication layers that reduce the interaction friction and enhance ion-pair mobility (Fig. 10i, j) [175]. Moreover, supramolecular design strategies integrating conductive polymers into flexible hydrogel matrices have been shown to improve conductivity, mechanical toughness, and self-healing capabilities. These multifunctional hydrogels enable the development of highly stretchable, biocompatible, and self-repairing wearable sensors [176, 177].

### Commentary and Comparison

The growth of dendrite, HER, and surface passivation have profound impacts on the discharge capacity, Coulombic efficiency, and cycling stability of AZIBs. To address these issues, constructing a thermally stable metal anode–electrolyte interface is crucial [178, 179]. Among various approaches, including anode structural modification and the introduction of interfacial catalysts, electrolyte engineering has proven to be one of the most effective approaches [180–182]. Based on this, several feasible optimization directions in electrolyte engineering can be proposed:

1. Zinc salt optimization: The selection of appropriate zinc salts can regulate electrolyte pH and chemical stability, thereby establishing a more favorable electrochemical environment. Additionally, concentration adjustment enables the expansion of the electrochemical stability window, contributing to enhanced AZIBs performance.
2. Electrolyte additives: Incorporating various additives, such as alkali metal ions for electrostatic shielding, organic molecules with strong adsorption capability, desolvation agents, SEI film precursors, and dynamic interfacial regulators, can significantly improve Zn^2+^ deposition behavior and suppress side reactions. Notably, these mechanisms often act synergistically rather than independently [183]. For instance, adsorption not only shortens the Zn^2+^ two-dimensional diffusion time, increases nucleation site density, and lowers the nucleation energy barrier to promote uniform and directional Zn^2+^ deposition, but also facilitates rapid interfacial passivation to form a robust artificial SEI layer. Therefore, additive design should comprehensively consider the interaction among these multiple functional mechanisms.
3. Development of advanced electrolytes: Emerging systems, including gel and hydrogel electrolytes with reduced free water content, offer enhanced stability and suppressed reactivity. These systems enable AZIBs to operate over a wide temperature range and at high current densities, making them particularly suitable for flexible and wearable electronic devices.

To aid comparison and provide clarity, Table 3 summarizes the electrolyte additives discussed in this review along with their corresponding electrochemical performance metrics.

**Table 3:** Electrolyte additives and their impact on battery performance

Additive	Device	Main mechanism	Cycling performance	References
LiCl	Zn||Zn	Self-healing electrostatic shield	> 1000 cycles at 100 mA cm⁻^2^ CE ≈ 99.9%	[100]
Rb_2_SO_4_	Zn||VO_2_	Self-healing electrostatic shield	71.6% capacity retention after 500 cycles at 5 A g⁻^1^	[105]
Ga(NO_3_)_3_	Zn||VO_2_	Self-healing electrostatic shield	122.9 mAh g⁻^1^ retained after 1500 cycles at 5 A g⁻^1^	[104]
Graphene Oxide (GO)	Zn||MnO_2_	Adsorption effects	93% capacity retention after 250 cycles at 1 A g⁻^1^	[108]
6-Aminohexanoic acid (6AA)	Zn||MnO_2_	Adsorption effects	> 97.5% CE over 200 cycles at 0.5 A g⁻^1^	[113]
SDBS (anionic surfactant)	Zn||MnO_2_	Adsorption Effects	85% capacity retention after 1000 cycles at 1 A g⁻^1^	[109]
Hyaluronic acid (HA)	Zn||Zn	Desolvation	2200 h at 1 mA cm⁻^2^	[121]
Hyaluronic acid (HA)	Zn||MnO_2_	Desolvation	61.4% retention after 1000 cycles at 1 A g⁻^1^	[121]
2-Propanol	Zn||Zn	Desolvation	500 h at 15 mA cm⁻^2^	[120]
Dodecyl sulfate (OSO_3_R⁻)	Zn||N-doped porous carbon	Desolvation	89.1% retention after 40 000 cycles at 5 A g⁻^1^	[122]
APA (amphiphilic polymer)	Zn||Zn	Desolvation	8800 h at 1 mA cm⁻^2^	[123]
PAA (polyacrylic acid)	Zn||Zn	Desolvation	3293 h at 5 mA cm⁻^2^	[124]
Zn(H_2_PO_4_)_2_	Zn||V₂O₅	SEI formation	nearly 100% CE at 0.8 A g^−1^ over 1000 cycles	[130]
trimethylethyl ammonium trifluoromethanesulfonate (Me_3_EtNOTf)	Zn||VOPO_4_	SEI formation	average CE of 99.9% at 2 A g^−1^ after 6000 cycles	[131]
Poly (N-vinylpyrrolidone-*co*-methyl acrylate) dipolymer	Zn||NVO	SEI formation	capacity retention of 94.33% for 60 days	[132]
sucrose biomolecule	Zn||NVO	SEI formation	73.1% capacity retention at 1 A g^−1^ over 670 cycles	[133]
N-[2-(3,4-dihydroxyphenyl)ethyl]-2-methylacrylamide	Zn||NVO	SEI formation	CE of 99.84% at 1 A g^−1^ after 1000 cycles	[137]
saccharin	Zn||MnO_2_	SEI formation	CE of 99.9% after 7500 cycles	[138]
positively charged chlorinated graphene quantum dot	Zn||LMO	SEI formation	capacity retention of 86% after 100 cycles	[139]
graphitic carbon nitride quantum dots	Zn||MnO_2_	SEI formation	nearly 100% CE over 500 cycles	[15]
SrTiO_3_	Zn||MnO_2_	Regulate crystal plane	specific capacity of 238 mA h g^−1^ after 200 cycles at 0.5 A g^−1^	[150]
polyethylene glycol	Zn||V₂O₅	Regulate crystal plane	capacity retention of 84% at 15 A *g*^−1^ after 500 cycles	[151]
Dextran(with a 70 000 molecular weight)	Zn||MnO_2_	Regulate crystal plane	capacity retention of 83% at 1 A *g*^−1^after 3000 cycles	[152]
2-methacryloyloxyethyl phosphorylcholine and N-acryloyl glycinamide	Zn||PANI	Regulate crystal plane	capacity retention of 90% at 15 A *g*^−1^ after 12,000 cycles	[153]
1-butyl-3-methylimidazolium cation	Zn||VOH	Regulate crystal plane	capacity retention of 90% at 0.2 A *g*^−1^ after 300 cycles	[154]

It is particularly noteworthy that the aforementioned electrolyte engineering strategies must be considered in tandem with their potential effects on cathode materials. As summarized in Table 3, the compatibility between electrolyte additives and cathode materials plays a critical role in the overall electrochemical performance of AZIBs. For instance, vanadium oxide cathodes are susceptible to dissolution, prompting the use of Zn(CF_3_SO_3_)_2_-based electrolytes to mitigate degradation. Vanadate-based cathodes often require the incorporation of metal ions to suppress active material leaching. Manganese-based cathodes frequently employ mixed electrolyte systems to alleviate Mn dissolution. For Prussian blue analogs (PBAs), controlling electrolyte concentration or introducing metal ion additives (such as Ni^2+^) is essential to prevent structural phase transitions and capacity fading [58, 184]. Therefore, future research should focus on the rational design of cathode–anode compatible electrolyte systems to achieve coordinated optimization of full cell performance.

## Summary and Prospects

This review highlights the electrolyte-related challenges associated with Zn metal anode in AZIBs and discusses corresponding strategies for their mitigation. Key issues, including dendrite growth, HER, and surface passivation, substantially compromise the electrochemical performance and safety of Zn metal anodes. Dendrite formation not only reduces the active anode surface area but also poses a risk of internal short circuits. Concurrently, HER and surface passivation induce energy loss and, in some cases, can cause battery swelling, posing serious safety concerns. The complexity and interconnectivity of these degradation mechanisms have prompted the development of diverse mitigation strategies, among which electrolyte structure engineering has emerged as one of the most effective.

Based on the above, viable approaches in electrolyte design include zinc salts optimization, the development of functional additives, and the design of novel electrolytes. However, several pressing challenges and emerging directions remain to be addressed:

1. Dynamic Reconstruction Techniques: Conventional stationary interfacial modification methods often fail to maintain long-term efficacy in complex operating environments due to continuous microstructural evolution and localized electrochemical heterogeneity. A dynamic and adaptive interfacial layer capable of real-time self-regulation is essential. Recent developments in dynamic self-healing and dynamic interface reconstruction technologies have shown promise in stabilizing the anode/electrolyte interface via autonomous structural reconfiguration during charging/discharging processes.
2. Artificial intelligence (AI)-Guided Additive Screening: The integration of AI enables high-throughput screening, property prediction, and intelligent materials discovery with minimal computational cost. Future research might focus on leveraging AI for efficient additive design and formulation optimization to meet specific requirements, such as reducing the nucleation barriers, controlling the nucleation rate, promoting Frank–Van der Merwe deposition, etc. These advancements will accelerate the development of next-generation AZIBs systems with enhanced interfacial control and electrochemical performance.
3. Adaptability to Extreme Conditions: Under harsh operating conditions, such as low temperatures and high current densities, the kinetics of ion transport and interfacial processes become increasingly complex. Tailoring electrolytes to ensure high-rate capability and robust cycling stability under such conditions remains a key direction for future development.
4. Application-specific Electrolyte Engineering: Beyond improving intrinsic performance, the electrolytes for AZIBs should be tailored for diverse application scenarios. Strategic electrolyte modifications can broaden AZIBs utility across fields, such as biodegradable electronics, implantable medical devices, and flexible/wearable systems, thereby facilitating cross-disciplinary innovation.

Despite considerable advancements in electrolyte structure regulation, unresolved challenges related to Zn anodes require more systematic and integrative solutions. Given the intrinsic coupling of dendrite growth, HER, and passivation, single-strategy approaches often face inherent limitations. Therefore, multi-functional and synergistic strategies that simultaneously improve performance, cost-efficiency, and scalability are urgently needed. In this context, a deeper understanding of interfacial mechanisms and ion transport kinetics is vital for enhancing electrochemical efficiency and prolonging battery lifespan. Furthermore, this review emphasizes the importance of developing electrolyte systems that are not only functionally robust and chemically stable but also environmentally sustainable and economically viable. Future research should incorporate comprehensive assessments of ecological impact and cost to guide the practical deployment of AZIBs. Given their potential in large-scale, sustainable energy storage, continued innovation in electrolyte structure design will be instrumental in positioning AZIBs as a leading solution for future clean energy technologies.

## References

[1] D.T. Boyle, W. Huang, H. Wang, Y. Li, H. Chen et al., Corrosion of lithium metal anodes during calendar ageing and its microscopic origins. Nat. Energy **6**(5), 487–494 (2021). 10.1038/s41560-021-00787-9

[2] Y. Zhang, E.H. Ang, K.N. Dinh, K. Rui, H. Lin et al., Recent advances in vanadium-based cathode materials for rechargeable zinc ion batteries. Mater. Chem. Front. **5**(2), 744–762 (2021). 10.1039/D0QM00577K

[3] S.-J. Yang, L.-L. Zhao, Z.-X. Li, P. Wang, Z.-L. Liu et al., Achieving stable Zn anode *via* artificial interfacial layers protection strategies toward aqueous Zn-ion batteries. Coord. Chem. Rev. **517**, 216044 (2024). 10.1016/j.ccr.2024.216044

[4] C.J.M. Melief, Smart delivery of vaccines. Nat. Mater. **17**(6), 482–483 (2018). 10.1038/s41563-018-0085-6 29795220 10.1038/s41563-018-0085-6

[5] Z. Cao, P. Zhuang, X. Zhang, M. Ye, J. Shen et al., Strategies for dendrite-free anode in aqueous rechargeable zinc ion batteries. Adv. Energy Mater. **10**(30), 2001599 (2020). 10.1002/aenm.202001599

[6] J. Song, K. Xu, N. Liu, D. Reed, X. Li, Crossroads in the renaissance of rechargeable aqueous zinc batteries. Mater. Today **45**, 191–212 (2021). 10.1016/j.mattod.2020.12.003

[7] Z. Cao, X. Zhu, D. Xu, P. Dong, M.O.L. Chee et al., Eliminating Zn dendrites by commercial cyanoacrylate adhesive for zinc ion battery. Energy Storage Mater. **36**, 132–138 (2021). 10.1016/j.ensm.2020.12.022

[8] H. Meng, Q. Ran, T.-Y. Dai, H. Shi, S.-P. Zeng et al., Surface-alloyed nanoporous zinc as reversible and stable anodes for high-performance aqueous zinc-ion battery. Nano-Micro Lett. **14**(1), 128 (2022). 10.1007/s40820-022-00867-9 10.1007/s40820-022-00867-9 PMC9198195 35699828

[9] Y. Ai, C. Yang, Z. Yin, T. Wang, T. Gai et al., Biomimetic superstructured interphase for aqueous zinc-ion batteries. J. Am. Chem. Soc. **146**(22), 15496–15505 (2024). 10.1021/jacs.4c03943 38785353 10.1021/jacs.4c03943

[10] S. Li, D. Yu, L. Liu, S. Yao, X. Wang et al., *In-situ* electrochemical induced artificial solid electrolyte interphase for MnO@C nanocomposite enabling long-lived aqueous zinc-ion batteries. Chem. Eng. J. **430**, 132673 (2022). 10.1016/j.cej.2021.132673

[11] L. Huang, J. Pu, Y. Zhao, X. Fang, Y. Yu et al., Phosphorus-doped carbon as an effective protective layer for advanced aqueous zinc-ion batteries. Chin. Chem. Lett. **36**(8), 110989 (2025). 10.1016/j.cclet.2025.110989

[12] J. Wei, P. Zhang, J. Sun, Y. Liu, F. Li et al., Advanced electrolytes for high-performance aqueous zinc-ion batteries. Chem. Soc. Rev. **53**(20), 10335–10369 (2024). 10.1039/d4cs00584h 39253782 10.1039/d4cs00584h

[13] G. Qu, H. Wei, S. Zhao, Y. Yang, X. Zhang et al., A temperature self-adaptive electrolyte for wide-temperature aqueous zinc-ion batteries. Adv. Mater. **36**(29), 2400370 (2024). 10.1002/adma.202400370 10.1002/adma.202400370 38684215

[14] Y. Zhang, F. Wan, S. Huang, S. Wang, Z. Niu et al., A chemically self-charging aqueous zinc-ion battery. Nat. Commun. **11**, 2199 (2020). 10.1038/s41467-020-16039-5 32366904 10.1038/s41467-020-16039-5 PMC7198488

[15] W. Zhang, M. Dong, K. Jiang, D. Yang, X. Tan et al., Self-repairing interphase reconstructed in each cycle for highly reversible aqueous zinc batteries. Nat. Commun. **13**(1), 5348 (2022). 10.1038/s41467-022-32955-0 36097022 10.1038/s41467-022-32955-0 PMC9468148

[16] L. Jiang, D. Li, X. Xie, D. Ji, L. Li et al., Electric double layer design for Zn-based batteries. Energy Storage Mater. **62**, 102932 (2023). 10.1016/j.ensm.2023.102932

[17] Z. Hu, F. Zhang, F. Wu, H. Wang, A. Zhou et al., Screening metal cation additives driven by differential capacitance for Zn batteries. Energy Environ. Sci. **17**(13), 4794–4802 (2024). 10.1039/d4ee01127a

[18] K. Guan, L. Tao, R. Yang, H. Zhang, N. Wang et al., Anti-corrosion for reversible zinc anode *via* a hydrophobic interface in aqueous zinc batteries. Adv. Energy Mater. **12**(9), 2103557 (2022). 10.1002/aenm.202103557

[19] Y. Chen, Z. Deng, Y. Sun, Y. Li, H. Zhang et al., Ultrathin zincophilic interphase regulated electric double layer enabling highly stable aqueous zinc-ion batteries. Nano-Micro Lett. **16**(1), 96 (2024). 10.1007/s40820-023-01312-1 10.1007/s40820-023-01312-1 PMC10810772 38270675

[20] H. Saboorian-Jooybari, Z. Chen, Calculation of re-defined electrical double layer thickness in symmetrical electrolyte solutions. Results Phys. **15**, 102501 (2019). 10.1016/j.rinp.2019.102501

[21] R. Zhao, H. Wang, H. Du, Y. Yang, Z. Gao et al., Lanthanum nitrate as aqueous electrolyte additive for favourable zinc metal electrodeposition. Nat. Commun. **13**(1), 3252 (2022). 10.1038/s41467-022-30939-8 35668132 10.1038/s41467-022-30939-8 PMC9170708

[22] Q. Zhang, J. Luan, Y. Tang, X. Ji, H. Wang, Interfacial design of dendrite-free zinc anodes for aqueous zinc-ion batteries. Angew. Chem. Int. Ed. **59**(32), 13180–13191 (2020). 10.1002/anie.202000162 10.1002/anie.202000162 32124537

[23] Y. Zhu, G. Liang, X. Cui, X. Liu, H. Zhong et al., Engineering hosts for Zn anodes in aqueous Zn-ion batteries. Energy Environ. Sci. **17**(2), 369–385 (2024). 10.1039/d3ee03584k

[24] C. Xie, Y. Li, Q. Wang, D. Sun, Y. Tang et al., Issues and solutions toward zinc anode in aqueous zinc-ion batteries: a mini review. Carbon Energy **2**(4), 540–560 (2020). 10.1002/cey2.67

[25] K. Zhao, G. Fan, J. Liu, F. Liu, J. Li et al., Boosting the kinetics and stability of Zn anodes in aqueous electrolytes with supramolecular cyclodextrin additives. J. Am. Chem. Soc. **144**(25), 11129–11137 (2022). 10.1021/jacs.2c00551 35700394 10.1021/jacs.2c00551

[26] R. Qin, Y. Wang, M. Zhang, Y. Wang, S. Ding et al., Tuning Zn^2+^ coordination environment to suppress dendrite formation for high-performance Zn-ion batteries. Nano Energy **80**, 105478 (2021). 10.1016/j.nanoen.2020.105478

[27] X. Zhou, Q. Zhang, Z. Hao, Y. Ma, O.A. Drozhzhin et al., Unlocking the allometric growth and dissolution of Zn anodes at initial nucleation and an early stage with atomic force microscopy. ACS Appl. Mater. Interfaces **13**(44), 53227–53234 (2021). 10.1021/acsami.1c16263 34699184 10.1021/acsami.1c16263

[28] Z. Hu, F. Zhang, A. Zhou, X. Hu, Q. Yan et al., Highly reversible Zn metal anodes enabled by increased nucleation overpotential. Nano-Micro Lett. **15**(1), 171 (2023). 10.1007/s40820-023-01136-z 10.1007/s40820-023-01136-z PMC10326211 37410259

[29] F. Lionetto, N. Arianpouya, B. Bozzini, A. Maffezzoli, M. Nematollahi et al., Advances in zinc-ion structural batteries. J. Energy Storage **84**, 110849 (2024). 10.1016/j.est.2024.110849

[30] D. Wang, W. Zhang, W. Zheng, X. Cui, T. Rojo et al., Towards high-safe lithium metal anodes: suppressing lithium dendrites *via* tuning surface energy. Adv. Sci. **4**(1), 1600168 (2017). 10.1002/advs.201600168 10.1002/advs.201600168 PMC5238744 28105393

[31] Y. Ma, Q. Ma, Y. Liu, Y. Tan, Y. Zhang et al., Multiphilic-Zn group “adhesion” strategy toward highly stable and reversible zinc anodes. Energy Storage Mater. **63**, 103032 (2023). 10.1016/j.ensm.2023.103032

[32] Z. Ye, S. Xie, Z. Cao, L. Wang, D. Xu et al., High-rate aqueous zinc-organic battery achieved by lowering *HOMO*/LUMO of organic cathode. Energy Storage Mater. **37**, 378–386 (2021). 10.1016/j.ensm.2021.02.022

[33] H. Zhang, F. Ning, Y. Guo, S. Subhan, X. Liu et al., Unraveling the mechanisms of aqueous zinc ion batteries *via* first-principles calculations. ACS Energy Lett. **9**(10), 4761–4784 (2024). 10.1021/acsenergylett.4c02014

[34] C. Liu, Z.G. Neale, G. Cao, Understanding electrochemical potentials of cathode materials in rechargeable batteries. Mater. Today **19**(2), 109–123 (2016). 10.1016/j.mattod.2015.10.009

[35] G. Xu, C. Pang, B. Chen, J. Ma, X. Wang et al., Prescribing functional additives for treating the poor performances of high-voltage (5 V-class) LiNi_0.5_Mn_1.5_O_4_/MCMB Li-ion batteries. Adv. Energy Mater. **8**(9), 1701398 (2018). 10.1002/aenm.201701398

[36] B. Li, Y. Chao, M. Li, Y. Xiao, R. Li et al., A review of solid electrolyte interphase (SEI) and dendrite formation in lithium batteries. Electrochem. Energy Rev. **6**(1), 7 (2023). 10.1007/s41918-022-00147-5

[37] S. Wang, Y. Ying, S. Chen, H. Wang, K.K.K. Cheung et al., Highly reversible zinc metal anode enabled by zinc fluoroborate salt-based *hydrous* organic electrolyte. Energy Storage Mater. **63**, 102971 (2023). 10.1016/j.ensm.2023.102971

[38] H. Tian, J.-L. Yang, Y. Deng, W. Tang, R. Liu et al., Steel anti-corrosion strategy enables long-cycle Zn anode. Adv. Energy Mater. **13**(1), 2202603 (2023). 10.1002/aenm.202202603

[39] T. Li, S. Hu, C. Wang, D. Wang, M. Xu et al., Engineering fluorine-rich double protective layer on Zn anode for highly reversible aqueous zinc-ion batteries. Angew. Chem. Int. Ed. **62**(51), e202314883 (2023). 10.1002/anie.202314883 10.1002/anie.202314883 37924309

[40] C. Li, G. Qu, X. Zhang, C. Wang, X. Xu, Electrode/electrolyte interfacial chemistry modulated by chelating effect for high-performance zinc anode. Energy Environ. Mater. **7**(3), e12608 (2024). 10.1002/eem2.12608

[41] T. Wei, L.-E. Mo, Y. Ren, H. Zhang, M. Wang et al., Non-sacrificial anionic surfactant with high *HOMO* energy level as a general descriptor for zinc anode. Energy Storage Mater. **70**, 103525 (2024). 10.1016/j.ensm.2024.103525

[42] P. Peljo, H.H. Girault, Electrochemical potential window of battery electrolytes: the *HOMO*–LUMO misconception. Energy Environ. Sci. **11**(9), 2306–2309 (2018). 10.1039/c8ee01286e

[43] N. Zhang, F. Cheng, Y. Liu, Q. Zhao, K. Lei et al., Cation-deficient spinel ZnMn_2_O_4_ cathode in Zn(CF_3_SO_3_)_2_ electrolyte for rechargeable aqueous Zn-ion battery. J. Am. Chem. Soc. **138**(39), 12894–12901 (2016). 10.1021/jacs.6b05958 27627103 10.1021/jacs.6b05958

[44] G. Kasiri, R. Trócoli, A. Bani Hashemi, F. La Mantia, An electrochemical investigation of the aging of copper hexacyanoferrate during the operation in zinc-ion batteries. Electrochim. Acta **222**, 74–83 (2016). 10.1016/j.electacta.2016.10.155

[45] N.S.V. Narayanan, B.V. Ashokraj, S. Sampath, Ambient temperature, zinc ion-conducting, binary molten electrolyte based on acetamide and zinc perchlorate: application in rechargeable zinc batteries. J. Colloid Interface Sci. **342**(2), 505–512 (2010). 10.1016/j.jcis.2009.10.034 19914628 10.1016/j.jcis.2009.10.034

[46] H. Li, L. Ma, C. Han, Z. Wang, Z. Liu et al., Advanced rechargeable zinc-based batteries: recent progress and future perspectives. Nano Energy **62**, 550–587 (2019). 10.1016/j.nanoen.2019.05.059

[47] Q. Zhang, Y. Ma, Y. Lu, X. Zhou, L. Lin et al., Designing anion-type water-free Zn^2+^ solvation structure for robust Zn metal anode. Angew. Chem. Int. Ed. **60**(43), 23357–23364 (2021). 10.1002/anie.202109682 10.1002/anie.202109682 34382322

[48] Y. Zhu, J. Yin, X. Zheng, A.-H. Emwas, Y. Lei et al., Concentrated dual-cation electrolyte strategy for aqueous zinc-ion batteries. Energy Environ. Sci. **14**(8), 4463–4473 (2021). 10.1039/d1ee01472b

[49] Y. Wu, N. Wang, H. Liu, R. Cui, J. Gu et al., Self-healing of surface defects on Zn electrode for stable aqueous zinc-ion batteries *via* manipulating the electrode/electrolyte interphases. J. Colloid Interface Sci. **629**, 916–925 (2023). 10.1016/j.jcis.2022.09.022 36150269 10.1016/j.jcis.2022.09.022

[50] C. Xu, B. Li, H. Du, F. Kang, Energetic zinc ion chemistry: the rechargeable zinc ion battery. Angew. Chem. Int. Ed. **51**(4), 933–935 (2012). 10.1002/anie.201106307 10.1002/anie.201106307 22170816

[51] S. Huang, J. Zhu, J. Tian, Z. Niu, Recent progress in the electrolytes of aqueous zinc-ion batteries. Chem. Eur. J. **25**(64), 14480–14494 (2019). 10.1002/chem.201902660 31407398 10.1002/chem.201902660

[52] F. Wang, O. Borodin, T. Gao, X. Fan, W. Sun et al., Highly reversible zinc metal anode for aqueous batteries. Nat. Mater. **17**(6), 543–549 (2018). 10.1038/s41563-018-0063-z 29662160 10.1038/s41563-018-0063-z

[53] F. Sun, Q. Tang, D.-E. Jiang, Theoretical advances in understanding and designing the active sites for hydrogen evolution reaction. ACS Catal. **12**(14), 8404–8433 (2022). 10.1021/acscatal.2c02081

[54] F. Zhang, T. Liao, Q. Zhou, J. Bai, X. Li et al., Advancements in ion regulation strategies for enhancing the performance of aqueous Zn-ion batteries. Mater. Sci. Eng. R. Rep. **165**, 101012 (2025). 10.1016/j.mser.2025.101012

[55] L. Dong, X. Ma, Y. Li, L. Zhao, W. Liu et al., Extremely safe, high-rate and ultralong-life zinc-ion hybrid supercapacitors. Energy Storage Mater. **13**, 96–102 (2018). 10.1016/j.ensm.2018.01.003

[56] G. Fang, J. Zhou, A. Pan, S. Liang, Recent advances in aqueous zinc-ion batteries. ACS Energy Lett. **3**(10), 2480–2501 (2018). 10.1021/acsenergylett.8b01426

[57] J. Zhou, L. Shan, Z. Wu, X. Guo, G. Fang et al., Investigation of V_2_O_5_ as a low-cost rechargeable aqueous zinc ion battery cathode. Chem. Commun. **54**(35), 4457–4460 (2018). 10.1039/c8cc02250j 10.1039/c8cc02250j 29652066

[58] Q. Meng, T. Yan, Y. Wang, X. Lu, H. Zhou et al., Critical design strategy of electrolyte engineering toward aqueous zinc-ion battery. Chem. Eng. J. **497**, 154541 (2024). 10.1016/j.cej.2024.154541

[59] X. Zeng, J. Mao, J. Hao, J. Liu, S. Liu et al., Electrolyte design for *in situ* construction of highly Zn^2+^-conductive solid electrolyte interphase to enable high-performance aqueous Zn-ion batteries under practical conditions. Adv. Mater. **33**(11), 2007416 (2021). 10.1002/adma.202007416 10.1002/adma.202007416 33576130

[60] T.C. Li, D. Fang, J. Zhang, M.E. Pam, Z.Y. Leong et al., Recent progress in aqueous zinc-ion batteries: a deep insight into zinc metal anodes. J. Mater. Chem. A **9**(10), 6013–6028 (2021). 10.1039/D0TA09111A

[61] D. Yuan, J. Zhao, H. Ren, Y. Chen, R. Chua et al., Anion texturing towards dendrite-free Zn anode for aqueous rechargeable batteries. Angew. Chem. Int. Ed. **60**(13), 7213–7219 (2021). 10.1002/anie.202015488 10.1002/anie.202015488 33381887

[62] Z. Zhao, J. Zhao, Z. Hu, J. Li, J. Li et al., Long-life and deeply rechargeable aqueous Zn anodes enabled by a multifunctional brightener-inspired interphase. Energy Environ. Sci. **12**(6), 1938–1949 (2019). 10.1039/C9EE00596J

[63] N. Patil, C. de la Cruz, D. Ciurduc, A. Mavrandonakis, J. Palma et al., An ultrahigh performance zinc-organic battery using poly(catechol) cathode in Zn(TFSI)_2_-based concentrated aqueous electrolytes. Adv. Energy Mater. **11**(26), 2100939 (2021). 10.1002/aenm.202100939

[64] Y. Gui, Y. Lei, B.A. Fan, Investigation on the effect of different mild acidic electrolyte on ZIBs electrode/electrolyte interface and the performance improvements with the optimized cathode. Front. Chem. **8**, 827 (2020). 10.3389/fchem.2020.00827 33195037 10.3389/fchem.2020.00827 PMC7645205

[65] G. Li, Z. Yang, Y. Jiang, C. Jin, W. Huang et al., Towards polyvalent ion batteries: a zinc-ion battery based on NASICON structured Na_3_V_2_(PO_4_)_3_. Nano Energy **25**, 211–217 (2016). 10.1016/j.nanoen.2016.04.051

[66] Y. Wang, W. Yan, X. Zhu, J. Li, Z. Li et al., Boosting performance of quasi-solid-state zinc ion batteries *via* zincophilic solubilization. Angew. Chem. Int. Ed. **64**(35), e202508556 (2025). 10.1002/anie.202508556 10.1002/anie.202508556 40503631

[67] C. Guan, F. Hu, X. Yu, H.-L. Chen, G.-H. Song et al., High performance of HNaV_6_O_16_·4H_2_O nanobelts for aqueous zinc-ion batteries with *in situ* phase transformation by Zn(CF_3_SO_3_)_2_ electrolyte. Rare Met. **41**(2), 448–456 (2022). 10.1007/s12598-021-01778-1

[68] L. Xu, Y. Zhang, J. Zheng, H. Jiang, T. Hu et al., Ammonium ion intercalated hydrated vanadium pentoxide for advanced aqueous rechargeable Zn-ion batteries. Mater. Today Energy **18**, 100509 (2020). 10.1016/j.mtener.2020.100509

[69] Y. Fan, X. Yao, G. Wang, Y. Xie, T. Wu et al., Interlayer spacing optimization combined with zinc-philic engineering fostering efficient Zn^2+^ storage of V_2_CT_x_ MXenes for aqueous zinc-ion batteries. Small **21**(10), 2408930 (2025). 10.1002/smll.202408930 10.1002/smll.202408930 39817878

[70] C. Ma, X. Wang, W. Lu, K. Yang, N. Chen et al., Dual-parasitic effect enables highly reversible Zn metal anode for ultralong 25,000 cycles aqueous zinc-ion batteries. Nano Lett. **24**(13), 4020–4028 (2024). 10.1021/acs.nanolett.4c00873 38517395 10.1021/acs.nanolett.4c00873

[71] X. Shi, J. Zeng, A. Yi, F. Wang, X. Liu et al., Unveiling the failure mechanism of Zn anodes in zinc trifluorosulfonate electrolyte: the role of micelle-like structures. J. Am. Chem. Soc. **146**(29), 20508–20517 (2024). 10.1021/jacs.4c07015 38996190 10.1021/jacs.4c07015

[72] X. Feng, P. Li, J. Yin, Z. Gan, Y. Gao et al., Enabling highly reversible Zn anode by multifunctional synergistic effects of hybrid solute additives. ACS Energy Lett. **8**(2), 1192–1200 (2023). 10.1021/acsenergylett.2c02455

[73] L. Zhang, I.A. Rodríguez-Pérez, H. Jiang, C. Zhang, D.P. Leonard et al., ZnCl_2_ “water-in-salt” electrolyte transforms the performance of vanadium oxide as a Zn battery cathode. Adv. Funct. Mater. **29**(30), 1902653 (2019). 10.1002/adfm.201902653

[74] D. Li, T. Sun, T. Ma, W. Zhang, Q. Sun et al., Regulating Zn^2+^ solvation shell through charge-concentrated anions for high Zn plating/stripping coulombic efficiency. Adv. Funct. Mater. **34**(44), 2405145 (2024). 10.1002/adfm.202405145

[75] D. Feng, Y. Jiao, P. Wu, Proton-reservoir hydrogel electrolyte for long-term cycling Zn/PANI batteries in wide temperature range. Angew. Chem. Int. Ed. **62**(1), e202215060 (2023). 10.1002/anie.202215060 10.1002/anie.202215060 36344437

[76] P. He, J. Liu, X. Zhao, Z. Ding, P. Gao et al., A three-dimensional interconnected V_6_O_13_ nest with a V^5+^-rich state for ultrahigh Zn ion storage. J. Mater. Chem. A **8**(20), 10370–10376 (2020). 10.1039/D0TA03165H

[77] Y. Shi, R. Wang, S. Bi, M. Yang, L. Liu et al., An anti-freezing hydrogel electrolyte for flexible zinc-ion batteries operating at −70 °C. Adv. Funct. Mater. **33**(24), 2214546 (2023). 10.1002/adfm.202214546

[78] C. Zhang, W. Shin, L. Zhu, C. Chen, J.C. Neuefeind et al., The electrolyte comprising more robust water and superhalides transforms Zn-metal anode reversibly and dendrite-free. Carbon Energy **3**(2), 339–348 (2021). 10.1002/cey2.70

[79] J. Chen, Z. Yan, K. Li, A. Hu, B. Yang et al., Regulating the relationship between Zn^2+^ and water molecules in electrolytes for aqueous zinc-based batteries. Battery Energy **3**(2), 20230063 (2024). 10.1002/bte2.20230063

[80] J. Cao, D. Zhang, X. Zhang, Z. Zeng, J. Qin et al., Strategies of regulating Zn^2+^ solvation structures for dendrite-free and side reaction-suppressed zinc-ion batteries. Energy Environ. Sci. **15**(2), 499–528 (2022). 10.1039/D1EE03377H

[81] B.W. Olbasa, F.W. Fenta, S.-F. Chiu, M.-C. Tsai, C.-J. Huang et al., High-rate and long-cycle stability with a dendrite-free zinc anode in an aqueous Zn-ion battery using concentrated electrolytes. ACS Appl. Energy Mater. **3**(5), 4499–4508 (2020). 10.1021/acsaem.0c00183

[82] C. Zhang, J. Holoubek, X. Wu, A. Daniyar, L. Zhu et al., A ZnCl_2_ water-in-salt electrolyte for a reversible Zn metal anode. Chem. Commun. **54**(100), 14097–14099 (2018). 10.1039/c8cc07730d 10.1039/c8cc07730d 30488907

[83] C. Wang, Z. Pei, Q. Meng, C. Zhang, X. Sui et al., Toward flexible zinc-ion hybrid capacitors with superhigh energy density and ultralong cycling life: the pivotal role of ZnCl_2_ salt-based electrolytes. Angew. Chem. Int. Ed. **60**(2), 990–997 (2021). 10.1002/anie.202012030 10.1002/anie.202012030 32969140

[84] L.E. Blanc, D. Kundu, L.F. Nazar, Scientific challenges for the implementation of Zn-ion batteries. Joule **4**(4), 771–799 (2020). 10.1016/j.joule.2020.03.002

[85] L. Li, S. Liu, W. Liu, D. Ba, W. Liu et al., Electrolyte concentration regulation boosting zinc storage stability of high-capacity K_0.486_V_2_O_5_ cathode for bendable quasi-solid-state zinc ion batteries. Nano-Micro Lett. **13**(1), 34 (2021). 10.1007/s40820-020-00554-7 10.1007/s40820-020-00554-7 PMC8187517 34138229

[86] X. Zhong, F. Wang, Y. Ding, L. Duan, F. Shi et al., Water-in-salt electrolyte Zn/LiFePO_4_ batteries. J. Electroanal. Chem. **867**, 114193 (2020). 10.1016/j.jelechem.2020.114193

[87] L. Liu, X. Jiang, X. Wang, X. Li, Y. Liu et al., Inhibiting the zinc anodes corrosion to achieve ultra-stable high temperature aqueous zinc-ion hybrid supercapacitors. J. Power Source **622**, 235368 (2024). 10.1016/j.jpowsour.2024.235368

[88] H. Zhang, X. Liu, B. Qin, S. Passerini, Electrochemical intercalation of anions in graphite for high-voltage aqueous zinc battery. J. Power Source **449**, 227594 (2020). 10.1016/j.jpowsour.2019.227594

[89] A. Clarisza, H.K. Bezabh, S.-K. Jiang, C.-J. Huang, B.W. Olbasa et al., Highly concentrated salt electrolyte for a highly stable aqueous dual-ion zinc battery. ACS Appl. Mater. Interfaces **14**(32), 36644–36655 (2022). 10.1021/acsami.2c09040 35927979 10.1021/acsami.2c09040

[90] J. Han, A. Mariani, A. Varzi, S. Passerini, Green and low-cost acetate-based electrolytes for the highly reversible zinc anode. J. Power Source **485**, 229329 (2021). 10.1016/j.jpowsour.2020.229329

[91] J. Han, A. Mariani, M. Zarrabeitia, Z. Jusys, R.J. Behm et al., Zinc-ion hybrid supercapacitors employing acetate-based water-in-salt electrolytes. Small **18**(31), 2201563 (2022). 10.1002/smll.202201563 10.1002/smll.202201563 35810459

[92] Z.A. Zafar, G. Abbas, K. Knizek, M. Silhavik, P. Kumar et al., Chaotropic anion based “water-in-salt” electrolyte realizes a high voltage Zn–graphite dual-ion battery. J. Mater. Chem. A **10**(4), 2064–2074 (2022). 10.1039/D1TA10122F

[93] G. Yang, J. Huang, X. Wan, B. Liu, Y. Zhu et al., An aqueous zinc-ion battery working at −50℃ enabled by low-concentration perchlorate-based chaotropic salt electrolyte. EcoMat **4**(2), e12165 (2022). 10.1002/eom2.12165

[94] W. Cheng, M. Zhao, Y. Lai, X. Wang, H. Liu et al., Recent advances in battery characterization using *in situ* XAFS, SAXS, XRD, and their combining techniques: from single scale to multiscale structure detection. Exploration **4**(1), 20230056 (2024). 10.1002/EXP.20230056 38854491 10.1002/EXP.20230056 PMC10867397

[95] X. Zhao, Y. Wang, C. Huang, Y. Gao, M. Huang et al., Tetraphenylporphyrin-based chelating ligand additive as a molecular sieving interfacial barrier toward durable aqueous zinc metal batteries. Angew. Chem. Int. Ed. **62**(46), e202312193 (2023). 10.1002/anie.202312193 10.1002/anie.202312193 37772347

[96] T. Xue, Y. Mu, Z. Zhang, J. Guan, J. Qiu et al., Enhanced zinc deposition and dendrite suppression in aqueous zinc-ion batteries *via* citric acid-aspartame electrolyte additives. Adv. Energy Mater. **15**(26), 2500674 (2025). 10.1002/aenm.202500674

[97] S. Yang, Y. Zhao, C. Zhi, Insights into the role of electrolyte additives for stable Zn anodes. Energy Mater. **5**(2), 500021 (2025). 10.20517/energymater.2024.169

[98] F. Ding, W. Xu, G.L. Graff, J. Zhang, M.L. Sushko et al., Dendrite-free lithium deposition *via* self-healing electrostatic shield mechanism. J. Am. Chem. Soc. **135**(11), 4450–4456 (2013). 10.1021/ja312241y 23448508 10.1021/ja312241y

[99] S. Bertolini, A. Delcorte, P. Venezuela, Understanding the self-healing electrostatic shield mechanism at the lithium–metal anode surface. Chem. Mater. **36**(17), 8477–8487 (2024). 10.1021/acs.chemmater.4c01601

[100] Y. Yuan, S.D. Pu, M.A. Pérez-Osorio, Z. Li, S. Zhang et al., Diagnosing the electrostatic shielding mechanism for dendrite suppression in aqueous zinc batteries. Adv. Mater. **36**(9), 2307708 (2024). 10.1002/adma.202307708 10.1002/adma.202307708 37879760

[101] Y. Xu, J. Zhu, J. Feng, Y. Wang, X. Wu et al., A rechargeable aqueous zinc/sodium manganese oxides battery with robust performance enabled by Na_2_SO_4_ electrolyte additive. Energy Storage Mater. **38**, 299–308 (2021). 10.1016/j.ensm.2021.03.019

[102] F. Wan, L. Zhang, X. Dai, X. Wang, Z. Niu et al., Aqueous rechargeable zinc/sodium vanadate batteries with enhanced performance from simultaneous insertion of dual carriers. Nat. Commun. **9**(1), 1656 (2018). 10.1038/s41467-018-04060-8 29695711 10.1038/s41467-018-04060-8 PMC5916908

[103] P. Wang, X. Xie, Z. Xing, X. Chen, G. Fang et al., Mechanistic insights of Mg^2+^-electrolyte additive for high-energy and long-life zinc-ion hybrid capacitors. Adv. Energy Mater. **11**(30), 2101158 (2021). 10.1002/aenm.202101158

[104] J. Cao, Y. Jin, H. Wu, Y. Yue, D. Zhang et al., Enhancing zinc anode stability with gallium ion-induced electrostatic shielding and oriented plating. Adv. Energy Mater. **15**(6), 2403175 (2025). 10.1002/aenm.202403175

[105] X. Zhang, J. Chen, H. Cao, X. Huang, Y. Liu et al., Efficient suppression of dendrites and side reactions by strong electrostatic shielding effect *via* the additive of Rb_2_SO_4_ for anodes in aqueous zinc-ion batteries. Small **19**(52), 2303906 (2023). 10.1002/smll.202303906 10.1002/smll.202303906 37649229

[106] Z. Peng, H. Yan, Q. Zhang, S. Liu, S.C. Jun et al., Stabilizing zinc anode through ion selection sieving for aqueous Zn-ion batteries. Nano Lett. **24**(30), 9137–9146 (2024). 10.1021/acs.nanolett.4c00693 39037888 10.1021/acs.nanolett.4c00693

[107] J. Yang, M. Qiu, M. Zhu, C. Weng, Y. Li et al., Biomacromolecule guiding construction of effective interface layer for ultra-stable zinc anode. Energy Storage Mater. **67**, 103287 (2024). 10.1016/j.ensm.2024.103287

[108] J. Abdulla, J. Cao, D. Zhang, X. Zhang, C. Sriprachuabwong et al., Elimination of zinc dendrites by graphene oxide electrolyte additive for zinc-ion batteries. ACS Appl. Energy Mater. **4**(5), 4602–4609 (2021). 10.1021/acsaem.1c00224

[109] W. Xie, K. Zhu, W. Jiang, H. Yang, M. Ma et al., Highly 002-oriented dendrite-free anode achieved by enhanced interfacial electrostatic adsorption for aqueous zinc-ion batteries. ACS Nano **18**(32), 21184–21197 (2024). 10.1021/acsnano.4c04181 39094098 10.1021/acsnano.4c04181

[110] F. Jing, L. Xu, Y. Shang, G. Chen, C. Lv et al., Interface engineering enabled by sodium dodecyl sulfonate surfactant for stable Zn metal batteries. J. Colloid Interface Sci. **669**, 984–991 (2024). 10.1016/j.jcis.2024.05.059 38759597 10.1016/j.jcis.2024.05.059

[111] L. Peng, X. Ren, Z. Liang, Y. Sun, Y. Zhao et al., Reversible proton co-intercalation boosting zinc-ion adsorption and migration abilities in bismuth selenide nanoplates for advanced aqueous batteries. Energy Storage Mater. **42**, 34–41 (2021). 10.1016/j.ensm.2021.07.015

[112] K. Xie, K. Ren, C. Sun, S. Yang, M. Tong et al., Toward stable zinc-ion batteries: use of a chelate electrolyte additive for uniform zinc deposition. ACS Appl. Energy Mater. **5**(4), 4170–4178 (2022). 10.1021/acsaem.1c03558

[113] S.-H. Huh, Y.J. Choi, S.H. Kim, J.-S. Bae, S.-H. Lee et al., Enabling uniform zinc deposition by zwitterion additives in aqueous zinc metal anodes. J. Mater. Chem. A **11**(36), 19384–19395 (2023). 10.1039/D3TA01943H

[114] T. Zhang, Y. Tang, S. Guo, X. Cao, A. Pan et al., Fundamentals and perspectives in developing zinc-ion battery electrolytes: a comprehensive review. Energy Environ. Sci. **13**(12), 4625–4665 (2020). 10.1039/d0ee02620d

[115] H. Du, R. Zhao, Y. Yang, Z. Liu, L. Qie et al., High-capacity and long-life zinc electrodeposition enabled by a self-healable and desolvation shield for aqueous zinc-ion batteries. Angew. Chem. Int. Ed. **61**(10), e202114789 (2022). 10.1002/anie.202114789 10.1002/anie.202114789 34939320

[116] L. Ding, L. Wang, J. Gao, T. Yan, H. Li et al., Facile Zn^2+^ desolvation enabled by local coordination engineering for long-cycling aqueous zinc-ion batteries. Adv. Funct. Mater. **33**(32), 2301648 (2023). 10.1002/adfm.202301648

[117] W. Ma, S. Wang, X. Wu, W. Liu, F. Yang et al., Tailoring desolvation strategies for aqueous zinc-ion batteries. Energy Environ. Sci. **17**(14), 4819–4846 (2024). 10.1039/d4ee00313f

[118] Y. Xie, Q. Dou, G. Li, Y. Chen, X. Yan, Regulating the solvation environment of hybrid electrolytes towards high-temperature zinc-ion storage. Energy Mater **5**(3), 500025 (2025). 10.20517/energymater.2024.183

[119] B. Song, Q. Lu, X. Wang, P. Xiong, Promoted de-solvation effect and dendrite-free Zn deposition enabled by *in-situ* formed interphase layer for high-performance zinc-ion batteries. Energy Mater. **5**(3), 500031 (2025). 10.20517/energymater.2024.182

[120] Q. Ma, R. Gao, Y. Liu, H. Dou, Y. Zheng et al., Regulation of outer solvation shell toward superior low-temperature aqueous zinc-ion batteries. Adv. Mater. **34**(49), 2207344 (2022). 10.1002/adma.202207344 10.1002/adma.202207344 36177699

[121] M. Qiu, P. Sun, G. Cui, W. Mai, Chaotropic polymer additive with ion transport tunnel enable dendrite-free zinc battery. ACS Appl. Mater. Interfaces **14**(36), 40951–40958 (2022). 10.1021/acsami.2c10517 36039409 10.1021/acsami.2c10517

[122] G. Guo, C. Ji, H. Mi, C. Yang, M. Li et al., Zincophilic anionic hydrogel electrolyte with interfacial specific adsorption of solvation structures for durable zinc ion hybrid supercapacitors. Adv. Funct. Mater. **34**(2), 2308405 (2024). 10.1002/adfm.202308405

[123] B. Niu, Z. Li, D. Luo, X. Ma, Q. Yang et al., Nano-scaled hydrophobic confinement of aqueous electrolyte by a nonionic amphiphilic polymer for long-lasting and wide-temperature Zn-based energy storage. Energy Environ. Sci. **16**(4), 1662–1675 (2023). 10.1039/D2EE04023A

[124] K. Ouyang, F. Li, D. Ma, Y. Wang, S. Shen et al., Trace-additive-mediated hydrophobic structure editing of aqueous zinc metal batteries for enabling all-climate long-term operation. ACS Energy Lett. **8**(12), 5229–5239 (2023). 10.1021/acsenergylett.3c01872

[125] D. Xie, Y. Sang, D.-H. Wang, W.-Y. Diao, F.-Y. Tao et al., ZnF_2_-riched inorganic/organic hybrid SEI: in situ-chemical construction and performance-improving mechanism for aqueous zinc-ion batteries. Angew. Chem. Int. Ed. **62**(7), e202216934 (2023). 10.1002/anie.202216934 10.1002/anie.202216934 36478517

[126] T. Zhao, H. Wu, X. Wen, J. Zhang, H. Tang et al., Recent advances in MOFs/MOF derived nanomaterials toward high-efficiency aqueous zinc ion batteries. Coord. Chem. Rev. **468**, 214642 (2022). 10.1016/j.ccr.2022.214642

[127] W. Wu, Y. Deng, G. Chen, A self-repairing polymer-inorganic composite coating to enable high-performance Zn anodes for zinc-ion batteries. Chin. Chem. Lett. **34**(12), 108424 (2023). 10.1016/j.cclet.2023.108424

[128] L. Yuan, J. Hao, B. Johannessen, C. Ye, F. Yang et al., Hybrid working mechanism enables highly reversible Zn electrodes. eScience **3**(2), 100096 (2023). 10.1016/j.esci.2023.100096

[129] H. Cheng, S. Zhang, W. Guo, Q. Wu, Z. Shen et al., Hydrolysis of solid buffer enables high-performance aqueous zinc ion battery. Adv. Sci. **11**(7), 2307052 (2024). 10.1002/advs.202307052 10.1002/advs.202307052 PMC10870042 38063837

[130] X. Zeng, J. Mao, J. Hao, J. Liu, S. Liu et al., Electrolyte design for *in situ* construction of highly Zn(2+)-conductive solid electrolyte interphase to enable high-performance aqueous Zn-ion batteries under practical conditions. Adv. Mater. **33**(11), e2007416 (2021). 10.1002/adma.202007416 33576130 10.1002/adma.202007416

[131] L. Cao, D. Li, T. Pollard, T. Deng, B. Zhang et al., Fluorinated interphase enables reversible aqueous zinc battery chemistries. Nat. Nanotechnol. **16**(8), 902–910 (2021). 10.1038/s41565-021-00905-4 33972758 10.1038/s41565-021-00905-4

[132] D. Luo, X. Ma, P. Du, Z. Chen, Q. Lin et al., Reconstructing solvation structure by steric hindrance-coordination push-pull of dipolymer-H_2_O-Zn^2+^ toward long-life aqueous zinc-metal batteries. Angew. Chem. Int. Ed. **63**(28), e202401163 (2024). 10.1002/anie.202401163 10.1002/anie.202401163 38702974

[133] H. Dou, X. Wu, M. Xu, R. Feng, Q. Ma et al., Steric-hindrance effect tuned ion solvation enabling high performance aqueous zinc ion batteries. Angew. Chem. Int. Ed. **63**(21), e202401974 (2024). 10.1002/anie.202401974 10.1002/anie.202401974 38470070

[134] H. Yin, H. Wu, Y. Yang, S. Yao, P. Han et al., Electrical double layer and *in situ* polymerization SEI enables high reversible zinc metal anode. Small **20**(50), 2404367 (2024). 10.1002/smll.202404367 10.1002/smll.202404367 39344599

[135] H. Wu, H. Yin, H. Tian, J. Yang, R. Liu, Stable Zn-metal anode enabled by solvation structure modulation and *in situ* SEI layer construction. Energy Environ. Mater. **8**(2), e12839 (2025). 10.1002/eem2.12839

[136] W. Shao, C. Li, C. Wang, G. Du, S. Zhao et al., Stabilization of zinc anode by trace organic corrosion inhibitors for long lifespan. Chin. Chem. Lett. **36**(3), 109531 (2025). 10.1016/j.cclet.2024.109531

[137] H. Wu, H.-T. Yin, J.-L. Yang, R. Liu, Chelation effect induced robust biomass protective layer for aqueous Zn metal anode. Adv. Energy Mater. **15**(30), 2501359 (2025). 10.1002/aenm.202501359

[138] C. Huang, X. Zhao, S. Liu, Y. Hao, Q. Tang et al., Stabilizing zinc anodes by regulating the electrical double layer with saccharin anions. Adv. Mater. **33**(38), 2100445 (2021). 10.1002/adma.202100445 10.1002/adma.202100445 34338350

[139] H. Wang, A. Zhou, X. Hu, Z. Hu, F. Zhang et al., Bifunctional dynamic adaptive interphase reconfiguration for zinc deposition modulation and side reaction suppression in aqueous zinc ion batteries. ACS Nano **17**(12), 11946–11956 (2023). 10.1021/acsnano.3c04155 37318040 10.1021/acsnano.3c04155

[140] S. Zhang, J. Li, B. Jin, M. Shao, Oriented zinc metal anode based on directional recognition and assembly. Small **19**(38), 2301874 (2023). 10.1002/smll.202301874 10.1002/smll.202301874 37196419

[141] L. Wang, Y. Shao, Z. Fu, X. Zhang, J. Kang et al., Synergistically enhancing the selective adsorption for crystal planes to regulate the (002)-texture preferred Zn deposition *via* supramolecular host–guest units. Energy Environ. Sci. **18**(10), 4859–4871 (2025). 10.1039/d5ee00763a

[142] Y. Lu, T. Wang, Z. Li, H. Cheng, K. Peng et al., Epitaxial deposition of Zn (002) for stable zinc metal anodes. Chem. Eng. J. **458**, 141509 (2023). 10.1016/j.cej.2023.141509

[143] M. Zhou, S. Guo, J. Li, X. Luo, Z. Liu et al., Surface-preferred crystal plane for a stable and reversible zinc anode. Adv. Mater. **33**(21), 2100187 (2021). 10.1002/adma.202100187 10.1002/adma.202100187 33864653

[144] X. Liu, Y. Guo, F. Ning, Y. Liu, S. Shi et al., Fundamental understanding of hydrogen evolution reaction on zinc anode surface: a first-principles study. Nano-Micro Lett. **16**(1), 111 (2024). 10.1007/s40820-024-01337-0 10.1007/s40820-024-01337-0 PMC11250978 38321305

[145] Z. Xing, C. Huang, Z. Hu, Advances and strategies in electrolyte regulation for aqueous zinc-based batteries. Coord. Chem. Rev. **452**, 214299 (2022). 10.1016/j.ccr.2021.214299

[146] M. Xi, Z. Liu, W. Wang, Z. Qi, R. Sheng et al., Shear-flow induced alignment of graphene enables the closest packing crystallography of the (002) textured zinc metal anode with high reversibility. Energy Environ. Sci. **17**(9), 3168–3178 (2024). 10.1039/D3EE04360F

[147] Y. Yan, C. Shu, T. Zeng, X. Wen, S. Liu et al., Surface-preferred crystal plane growth enabled by underpotential deposited monolayer toward dendrite-free zinc anode. ACS Nano **16**(6), 9150–9162 (2022). 10.1021/acsnano.2c01380 35696327 10.1021/acsnano.2c01380

[148] H.B. Jeong, D.I. Kim, G. Yoo, D. Mohan, A. Roy et al., Selective control of sharp-edge zinc electrodes with (002) plane for high-performance aqueous zinc-ion batteries. J. Mater. Chem. A **12**(25), 15265–15277 (2024). 10.1039/D4TA01013B

[149] K.E.K. Sun, T.K.A. Hoang, T.N.L. Doan, Y. Yu, P. Chen, Highly sustainable zinc anodes for a rechargeable hybrid aqueous battery. Chem. Eur. J. **24**(7), 1667–1673 (2018). 10.1002/chem.201704440 29152794 10.1002/chem.201704440

[150] R. Deng, Z. He, F. Chu, J. Lei, Y. Cheng et al., An aqueous electrolyte densified by perovskite SrTiO(3) enabling high-voltage zinc-ion batteries. Nat. Commun. **14**(1), 4981 (2023). 10.1038/s41467-023-40462-z 37591851 10.1038/s41467-023-40462-z PMC10435537

[151] Y. Wu, Z. Zhu, D. Shen, L. Chen, T. Song et al., Electrolyte engineering enables stable Zn-ion deposition for long-cycling life aqueous Zn-ion batteries. Energy Storage Mater. **45**, 1084–1091 (2022). 10.1016/j.ensm.2021.11.003

[152] J. Yang, Z. Ji, M. Deng, C. Weng, X. Wang et al., Chain-length engineered interfacial architecture enables dendrite-free aqueous zinc-ion batteries. Mater. Horiz. **12**(16), 6383–6394 (2025). 10.1039/d5mh00668f 40462580 10.1039/d5mh00668f

[153] D. Feng, Y. Jiao, P. Wu, Guiding Zn uniform deposition with polymer additives for long‐lasting and highly utilized Zn metal anodes. Angew. Chem. Int. Ed. **62**(51), e202314456 (2023). 10.1002/anie.202314456 10.1002/anie.202314456 37929923

[154] G. Ma, W. Yuan, X. Li, T. Bi, L. Niu et al., Organic cations texture zinc metal anodes for deep cycling aqueous zinc batteries. Adv. Mater. **36**(35), 2408287 (2024). 10.1002/adma.202408287 10.1002/adma.202408287 38967293

[155] C. Li, X. Xie, S. Liang, J. Zhou, Issues and future perspective on zinc metal anode for rechargeable aqueous zinc-ion batteries. Energy Environ. Mater. **3**(2), 146–159 (2020). 10.1002/eem2.12067

[156] W. Yang, X. Du, J. Zhao, Z. Chen, J. Li et al., Hydrated eutectic electrolytes with ligand-oriented solvation shells for long-cycling zinc-organic batteries. Joule **4**(7), 1557–1574 (2020). 10.1016/j.joule.2020.05.018

[157] D. Kumar, L.R. Franco, N. Abdou, R. Shu, A. Martinelli et al., Water-in-polymer salt electrolyte for long-life rechargeable aqueous zinc-lignin battery. Energy Environ. Mater. **8**(1), e12752 (2025). 10.1002/eem2.12752

[158] Z. Khan, D. Kumar, X. Crispin, Does water-in-salt electrolyte subdue issues of Zn batteries? Adv. Mater. **35**(36), 2300369 (2023). 10.1002/adma.202300369 10.1002/adma.202300369 37220078

[159] Z. Ali Zafar, G. Abbas, K. Knizek, M. Silhavik, P. Kumar et al., Chaotropic anion based “water-in-salt” electrolyte realizes a high voltage Zn–graphite dual-ion battery. J. Mater. Chem. A **10**(4), 2064–2074 (2022). 10.1039/D1TA10122F

[160] Y. Shen, B. Liu, X. Liu, J. Liu, J. Ding et al., Water-in-salt electrolyte for safe and high-energy aqueous battery. Energy Storage Mater. **34**, 461–474 (2021). 10.1016/j.ensm.2020.10.011

[161] R. Chen, C. Zhang, J. Li, Z. Du, F. Guo et al., A hydrated deep eutectic electrolyte with finely-tuned solvation chemistry for high-performance zinc-ion batteries. Energy Environ. Sci. **16**(6), 2540–2549 (2023). 10.1039/D3EE00462G

[162] L. Jiang, L. Yao, G. Wang, C. Liu, X. Chi et al., Long-duration aqueous Zn-ion batteries achieved by dual-salt highly-concentrated electrolyte with low water activity. J. Energy Chem. **101**, 778–785 (2025). 10.1016/j.jechem.2024.09.060

[163] J. Xie, D. Lin, H. Lei, S. Wu, J. Li et al., Electrolyte and interphase engineering of aqueous batteries beyond “water-in-salt” strategy. Adv. Mater. **36**(17), e2306508 (2024). 10.1002/adma.202306508 37594442 10.1002/adma.202306508

[164] J. Li, H. Zhang, Z. Liu, H. Du, H. Wan et al., Boosting dendrite-free zinc anode with strongly polar functional group terminated hydrogel electrolyte for high-safe aqueous zinc-ion batteries. Adv. Funct. Mater. **35**(2), 2412865 (2025). 10.1002/adfm.202412865

[165] R. Qi, W. Tang, Y. Shi, K. Teng, Y. Deng et al., Gel polymer electrolyte toward large-scale application of aqueous zinc batteries. Adv. Funct. Mater. **33**(47), 2306052 (2023). 10.1002/adfm.202306052

[166] Z. Zeng, S. Liao, G. Ma et al., High-conductivity and ultrastretchable self-healing hydrogels for flexible zinc-ion batteries. ACS Appl. Mater. Interfaces **16**(43), 58961–58972 (2024). 10.1021/acsami.4c13058 39432458 10.1021/acsami.4c13058

[167] Y. Hao, D. Feng, L. Hou, T. Li, Y. Jiao et al., Gel electrolyte constructing Zn (002) deposition crystal plane toward highly stable Zn anode. Adv. Sci. **9**(7), 2104832 (2022). 10.1002/advs.202104832 10.1002/advs.202104832 PMC8895127 35043600

[168] Y. Tang, C. Liu, H. Zhu, X. Xie, J. Gao et al., Ion-confinement effect enabled by gel electrolyte for highly reversible dendrite-free zinc metal anode. Energy Storage Mater. **27**, 109–116 (2020). 10.1016/j.ensm.2020.01.023

[169] S. Li, X. Fan, X. Liu, Z. Zhao, W. Xu et al., Potassium polyacrylate-based gel polymer electrolyte for practical Zn–Ni batteries. ACS Appl. Mater. Interfaces **14**(20), 22847–22857 (2022). 10.1021/acsami.1c20999 35103471 10.1021/acsami.1c20999

[170] M. Chen, J. Chen, W. Zhou, X. Han, Y. Yao et al., Realizing an all-round hydrogel electrolyte toward environmentally adaptive dendrite-free aqueous Zn–MnO_2_ batteries. Adv. Mater. **33**(9), 2007559 (2021). 10.1002/adma.202007559 10.1002/adma.202007559 33511697

[171] Q. He, G. Fang, Z. Chang, Y. Zhang, S. Zhou et al., Building ultra-stable and low-polarization composite Zn anode interface *via* hydrated polyzwitterionic electrolyte construction. Nano-Micro Lett. **14**(1), 93 (2022). 10.1007/s40820-022-00835-3 10.1007/s40820-022-00835-3 PMC8986915 35384517

[172] Q. Deng, W. Zhou, H. Wang, Q. Ma, C. Li et al., Design of a polymer electrolyte membrane for enhanced zinc anode stability in reversible aqueous zinc-ion batteries. Energy Mater. **5**(9), 500103 (2025). 10.20517/energymater.2024.299

[173] L. Sun, Y. Yao, L. Dai, M. Jiao, B. Ding et al., Sustainable and high-performance Zn dual-ion batteries with a hydrogel-based water-in-salt electrolyte. Energy Storage Mater. **47**, 187–194 (2022). 10.1016/j.ensm.2022.02.012

[174] P. Samanta, S. Ghosh, H. Kolya, C.-W. Kang, N.C. Murmu et al., Molecular crowded ″water-in-salt″ polymer gel electrolyte for an ultra-stable Zn-ion battery. ACS Appl. Mater. Interfaces **14**(1), 1138–1148 (2022). 10.1021/acsami.1c21189 34932312 10.1021/acsami.1c21189

[175] Y. Wang, Q. Li, H. Hong, S. Yang, R. Zhang et al., Lean-water hydrogel electrolyte for zinc ion batteries. Nat. Commun. **14**, 3890 (2023). 10.1038/s41467-023-39634-8 37393327 10.1038/s41467-023-39634-8 PMC10314915

[176] Z. Sun, Q. Ou, C. Dong, J. Zhou, H. Hu et al., Conducting polymer hydrogels based on supramolecular strategies for wearable sensors. Exploration **4**(5), 20220167 (2024). 10.1002/EXP.20220167 39439497 10.1002/EXP.20220167 PMC11491309

[177] M. Xu, J. Liao, J. Li, Y. Shi, Z. Zhang et al., Elastic nanoparticle-reinforced, conductive structural color hydrogel with super stretchability, self-adhesion, self-healing as electrical/optical dual-responsive visual electronic skins. Exploration **5**(2), 270008 (2025). 10.1002/EXP.70008 40395753 10.1002/EXP.70008 PMC12087393

[178] L. Sun, B. Zheng, W. Liu, Constructing high-throughput and highly adsorptive lithium–sulfur battery separator coatings based on three-dimensional hexagonal star-shaped MOF derivatives. J. Colloid Interface Sci. **679**, 197–205 (2025). 10.1016/j.jcis.2024.09.208 39362144 10.1016/j.jcis.2024.09.208

[179] W. Liu, C. Li, D. Li, G. Qu, M. Kong et al., Constructing zinc-tin alloy interface for highly stable alkaline zinc anode. Chin. Chem. Lett. **36**(7), 110152 (2025). 10.1016/j.cclet.2024.110152

[180] Y. Zhang, Z. Hu, Y. Bi et al., Cold-pressing strategy for constructing simple and high-performance dendrite-free zinc anodes for aqueous zinc-ion batteries. ACS Sustain. Chem. Eng. **13**(14), 5381–5393 (2025). 10.1021/acssuschemeng.5c00832

[181] J. Chen, L. Ren, X. Chen, Q. Wang, C. Chen et al., Well-defined nanostructures of high entropy alloys for electrocatalysis. Exploration **5**(2), 20230036 (2025). 10.1002/EXP.20230036 40395756 10.1002/EXP.20230036 PMC12087413

[182] J. Wang, C.-F. Du, Y. Xue, X. Tan, J. Kang et al., MXenes as a versatile platform for reactive surface modification and superior sodium-ion storages. Exploration **1**(2), 20210024 (2021). 10.1002/EXP.20210024 37323210 10.1002/EXP.20210024 PMC10191007

[183] X. Chen, P. Gao, W. Li, N.A. Thieu, Z.M. Grady et al., Stabilizing Zn anodes by molecular interface engineering with amphiphilic triblock copolymer. ACS Energy Lett. **9**(4), 1654–1665 (2024). 10.1021/acsenergylett.3c02824

[184] B. Ye, F. Wu, R. Zhao, H. Zhu, M. Lv et al., Electrolyte regulation toward cathodes with enhanced-performance in aqueous zinc ion batteries. Adv. Mater. **37**(15), 2501538 (2025). 10.1002/adma.202501538 10.1002/adma.202501538 40033963

